# The miR-183/96/182 Cluster Regulates the Functions of Corneal Resident Macrophages

**DOI:** 10.4049/immunohorizons.2000091

**Published:** 2020-11-18

**Authors:** Ardian Coku, Sharon A. McClellan, Eric Van Buren, Jessica B. Back, Linda D. Hazlett, Shunbin Xu

**Affiliations:** * Department of Ophthalmology, Visual and Anatomical Sciences, Wayne State University, Detroit, MI 48201;; † Department of Oncology, School of Medicine, Wayne State University, Detroit, MI 48201

## Abstract

Tissue-resident macrophages (ResMϕ) play important roles in the normal development and physiological functions as well as tissue repair and immune/inflammatory response to both internal and external insults. In cornea, ResMϕ are critical to the homeostasis and maintenance, wound healing, ocular immune privilege, and immune/inflammatory response to injury and microbial infection. However, the roles of microRNAs in corneal ResMϕ are utterly unknown. Previously, we demonstrated that the conserved miR-183/96/182 cluster (miR-183/96/182) plays important roles in sensory neurons and subgroups of both innate and adaptive immune cells and modulates corneal response to bacterial infection. In this study, we provide direct evidence that the mouse corneal ResMϕ constitutively produce both IL-17f and IL-10. This function is regulated by miR-183/96/182 through targeting Runx1 and Maf, key transcriptional regulators for IL-17f and IL-10 expression, respectively. In addition, we show that miR-183/96/182 has a negative feedback regulation on the TLR4 pathway in mouse corneal ResMϕ. Furthermore, miR-183/96/182 regulates the number of corneal ResMϕ. Inactivation of miR-183/96/182 in mouse results in more steady-state corneal resident immune cells, including ResMϕ, and leads to a simultaneous early upregulation of innate IL-17f and IL-10 production in the cornea after *Pseudomonas aeruginosa* infection. Its multiplex regulations on the simultaneous production of IL-17f and IL-10, TLR4 signaling pathway and the number of corneal ResMϕ place miR-183/96/182 in the center of corneal innate immunity, which is key to the homeostasis of the cornea, ocular immune privilege, and the corneal response to microbial infections.

## INTRODUCTION

Tissue-resident immune cells take residence in peripheral tissues early in development and become an integral part of their host tissues (1–3). Tissue-specific niches control their numbers and confer their tissue-specific identity. In addition to their roles as immune sentinels against pathogens, allergens, and tissue damage, tissue-resident immune cells play diverse and important roles in tissue development and homeostasis and repair and remodeling after defensive immune/inflammatory responses (1–3). In the eye, unique anatomical, physiological, and immunological features of the ocular surface work in concert to limit corneal inflammation and endow the eye with immune privilege (4). Besides the epithelium, endothelium, and stromal keratinocytes, corneal resident immune cells (CRICs) of hematopoietic origin reside in the cornea. Examples of CRICs include Langerhans cells (5–7), subsets of γδ T cells in the epithelial layer (8, 9), and monocytes (MC)/macrophages (Mϕ) and dendritic cells (DCs) in the stroma (5, 6, 10, 11). CRICs are considered to be important in the homeostasis of the cornea, for example, ocular immune privilege, transplant graft survival, wound healing, corneal nerve regeneration, inflammatory/immune responses following tissue damage and microbial infection, and orchestrate adaptive immune responses (4, 8, 9, 12–17). Despite their importance, a significant deficit remains in our understanding of CRICs.

In this regard, microRNAs (miRNAs) are small, noncoding regulatory RNAs and constitute a newly recognized, important mechanism of gene-expression regulation at posttranscriptional levels (18). Despite their identified roles in human diseases and potential as new therapeutic targets, the functions of miRNAs regarding CRICs remain unknown. Previously, we identified a conserved, paralogous miRNA cluster, the miR-183/96/182 cluster (miR-183/96/182), which encodes miR-183, miR-96, and miR-182 and is required for the normal development and function of all major sensory organs (19–21). Furthermore, we discovered that this miRNA cluster is also expressed and plays important roles in both innate and adaptive immune cells, such as Mϕ and neutrophils (22, 23) and Th17 cells (24), respectively. Inactivation of miR-183/96/182 in mice decreases the inflammatory response and reduces the severity of *Pseudomonas aeruginosa-induced* keratitis (22,23) and experimental autoimmune encephalomyelitis (24). We showed that miR-183/96/182 regulates the functions of Mϕ and neutrophils. Inactivation or knockdown of miR-183/96/182 resulted in increased phagocytosis and intracellular bacterial killing by Mϕ or neutrophils, a decreased production of proinflammatory cytokines by Mϕ (22, 23). In adaptive immunity, miR-183/96/182 promotes pathogenicity of Th17 cell pathogenicity (24).

Based on the above evidence, we hypothesize that miR-183/96/182 has a direct regulation on corneal resident innate immunity and the innate IL-17 response to *P. aeruginosa* infection. To test this hypothesis, we interrogated early (3 h postinfection [hpi]) corneal immune/inflammatory responses to *P. aeruginosa* infection in miR-183/96/182 knockout (ko) and wild-type (wt) control mice. Additionally, we evaluated the changes in the number and functional characteristics of CRICs as a result of inactivation of miR-183/96/182. In this study, we provide evidence that miR-183/96/182 regulates CRICs, including resident Mϕ (ResMϕ), and modulates the corneal innate IL-17 response to bacterial infection.

## MATERIALS AND METHODS

### Mice

All experiments and procedures involving animals and their care were pre-reviewed and approved by the Wayne State University Institutional Animal Care and Use Committee and carried out in accordance with National Institutes of Health and Association for Research in Vision and Ophthalmology guidelines. The miR-183/96/183 ko, the miR-183C^GT/GT^ mice, are on a 129S2/BL6-mixed background (20) and were originally derived from a gene-trap (GT) embryonic stem cell clone (20, 25, 26). Csf1r-EGFP or MacGreen mice (27) were purchased from The Jackson Laboratory [stock no. 018549, official name: B6N.Cg-Tg(Csf1r-EGFP) 1Hume/J]. The Csf1r-EGFP mice were bred with miR-183C^GT/+^ to produce miR-183/96/182 ko [Csf1r-EGFP(+);miR-183C^GT/GT^] and wt mice [Csf1r-EGFP(+);miR-183C^+/+^] as well as heterozygous mice [Csf1r-EGFP(+);miR-183C^GT/+^] on the background of Csf1r-EGFP with a normal mendelian inheritance pattern. Both male and female, age-matched, postnatal 21-d-old (P21), and young adult (8–12 wk old) mice were used in this study. The age of the mice in each experiment is specified in the figure legends and/or the text.

### Corneal infection

*P. aeruginosa* infection of the cornea was performed as described before (22). Briefly, mice were anesthetized with ethyl ether in a well-ventilated hood. The cornea of the left eye was wounded; 5.0 × 10^6^ CFU *P. aeruginosa* (strain 19660; American Type Culture Collection [ATCC], Manassas, VA) in a 5-μl vol was topically delivered. At 3 and 6 hpi, animals were euthanized, and corneas were harvested for RNA, protein lysate, or corneal flatmount preparation.

### RNA preparation, low-density gene-expression array screening, and real-time RT-PCR

Total RNA was prepared using RNeasy (QIAGEN, Germantown, MD) for mRNA studies as described previously (22). Custom-made RT^2^ Profiler PCR arrays (QIAGEN) were made for quick gene-expression analysis of IL-17 family cytokines and related genes following the manufacturer’s protocol. The initial custom array (catalog no. 330171 CLAIM25224; QIAGEN) was modified from the Th17 Response RT2 Profiler PCR array, including 88 proinflammatory cytokines and related genes on a 96-well plate (Supplemental Table I). After the initial screening, a focused custom array was designed (catalog no. 330171, CLAM30394; QIAGEN) to focus on the 19 dysregulated genes in the initial screening (Supplemental Table II). Subsequently, individual quantitative RT-PCR (qRT-PCR) assays were performed for confirmation using the QuantiFast SYBR Green RT-PCR kit and QuantiTect primers (QIAGEN) with β-actin or 18s rRNA as endogenous controls as described before (22).

### ELISA

Protein levels for IL-1β and Cxcl2 were tested using ELISA kits (R&D Systems, Minneapolis, MN) as described before (22). Briefly, corneas were homogenized in 1 ml of PBS with 0.1% Tween 20 and protease inhibitors (Roche Diagnostics, Indianapolis, IN). An aliquot of each supernatant was assayed in duplicate per the manufacturer’s instructions. Sensitivities of the ELISA assays were <2.31 pg/ml for IL-1β and <1.5 pg/ml for Cxcl2.

### Immunofluorescence

#### Abs.

Rat anti-mouse CD45 (clone 30-F11, catalog no. 5505539, 1/400 dilution), CD11b (clone M1/70, catalog no. 550282, 1/400 dilution), and hamster anti-mouse CD11c (clone HL3, catalog no. 550283, 1/50 dilution) were purchased from BD Biosciences (San Jose, CA); F4/80 (clone CI:A3–1, catalogno. MCA497R, 1/400 dilution) and from Bio-Rad Laboratories (Hercules, CA). Goat anti-rat IgG-Alexa Fluor 546 (AF546) (catalog no. A11081, 1/1000 dilution) and goat anti-hamster IgG-AF546 (catalog no. A21111, 1/1000 dilution) were purchased from Thermo Fisher Scientific (Grand Island, NY).

#### Flatmount cornea and corneal stroma preparation and immunofluorescence.

To avoid the disappearance of the CRICs during the dissection, we developed a method with minimal touch on the cornea. Briefly, mice were euthanized; eyes were enucleated and transferred to cold PBS. Under a dissecting scope (VWR International, Radnor, PA), a hole is punched through the sclera of the eye using a 19-gauge needle. Then, one blade of a pair of iris scissors was inserted into the hole to make a vertical cut to the cornea immediately anterior to the limbus. Thereafter, a circular cut was made on the cornea immediately anterior to the limbal region in parallel to the limbus. During the entire procedure, holding of the eyeball was on the sclera, whereas the cornea anterior to the circular cut was untouched. To prepare the stromal flatmount, the corneas were incubated in 20 mM EDTA in PBS at 37°C for 40 min. Subsequently, the epithelial layer was peeled away from the stroma. The corneas or both epithelial and stromal layers of the cornea were transferred to cold 1% paraformaldehyde (PFA) in 0.1 M phosphate buffer (PB), pH 7.4 for 1 h at 4°C. For direct confocal microscopy, the cornea or the stromal and epithelial layers of the cornea were flattened by four to six evenly spaced cuts from the periphery toward the center and mounted in VECTA-SHIELD media with DAPI (Vector Laboratories, Burlingame, CA) on Superfrost Plus slides (Fisherbrand). For immunofluorescence, after fixation, the corneas were incubated in a blocking buffer with 2% normal goat serum (Vector Laboratories) in PBS plus 2 mM MgCl_2_ (PBS+) for 30 min at room temperature (RT), then permeabilized with 0.1% Triton X-100 in the blocking buffer for 30 min at RT. Subsequently, corneal tissues were incubated with primary Abs in the blocking buffer overnight at 4°C. After four washes in PBS+ for 10 min at RT, the corneal tissues were incubated with AF546-conjugated secondary Abs (Thermo Fisher Scientific) for 2 h at RT. After four washes in PBS+, the corneal tissues were flattened and mounted on slides. All slides were studied and imaged using a TCS SP8 laser confocal microscope (Leica Microsystems, Buffalo Grove, IL). Negative controls were treated similarly with omission of primary Ab.

#### Confocal imaging and cell counting of flatmount cornea.

The entire cornea or corneal stroma was analyzed in all samples. To do so, series of Z-stacked images under 10× objective were taken across the entire cornea and stitched together to produce images of entire cornea at all planes. These stitched Z-stacked images were merged manually to reveal all EGFP^+^ cells in the cornea (Adobe Photoshop CS6). Cell counting was performed using ImageJ 1.52p software (http://imagej.nih.gov/ij; National Institutes of Health, Bethesda, MD).

#### Corneal cross-section preparation and immunofluorescence.

A similar protocol was followed as we described previously (20) with modifications. Briefly, mice were anesthetized with ketamine (80 mg/ml) and xylazine (16 mg/ml; Butler Schein, Columbus, OH) and perfused with PBS for 15 min, followed by 4% PFA in 0.1 M phosphate buffer (pH 7.4) for 20 min. Subsequently, the eyes were carefully enucleated and postfixed in 4% PFA in 0.1 M phosphate buffer for 1 h at 4°C and then soaked in 20% sucrose in PBS overnight at 4°C. Then, the eyes were mounted in optimum cutting temperature (Fisher Healthcare, Houston, TX). Twelve-micrometer, cryoprotected cross-sections were made for immunofluorescence as described previously (20).

### FACS

A similar protocol as described before (17) was followed with modifications. Briefly, corneas anterior to the limbus were excised, pooled, and incubated in HBSS with 0.2% collagenase A (catalog no. 10 103 578 001; Sigma-Aldrich. St. Louis, MO). Corneas were triturated to a single-cell suspension using a P1000 pipette for ~20 times and a small-bore hole glass pipette for ~50 times in DMEM with 5% FBS and 20 mM 2-ME (Sigma-Aldrich) and then filtered through a 70-μm filter (Miltenyi Biotec, Gaithersburg, MD). Cells were washed and resuspended in FACS buffer (DMEM medium with 10 mM HEPES and 1% FBS) containing 20 μg of Fc blocking Ab (purified anti-mouse CD16/32 [clone 93; eBioscience, Thermo Fisher Scientific] and anti-mouse CD16.2 [FcγRIV] Abs [clone 9E9; BioLegend, San Diego, CA]) and incubated on ice for 10 min. After blocking, cells were stained with an Ab mixture for 30 min on ice. The Ab mixture includes APC/fire750-CD45 (clone 30-F11; BioLegend), BUV395-CD11b (clone M1/70; BD Biosciences), PE-F4/80 (clone BM8; eBioscience), and PE/Dazzle594-CD11c (clone 223H7; BioLegend) in BD Horizon Brilliant Stain Buffer (BD Biosciences). After washing with FACS buffer, Ab-stained cells were incubated with Fixable Viability Dye eFluor 450 (eBio-science) for 30 min on ice. After washing, cell profiles were analyzed on a Sony SY3200 cell sorter at the Microscopy, Imaging and Cytometry Resources Core, Wayne State University. BD CompBeads (BD Biosciences) were used to optimize fluorescence compensation settings for multicolor flow cytometry. Cells incubated with isotype-matched control Abs served as negative controls. CD45^+^CD11b^+^F4/80^+^ cells were sorted, and total RNA was prepared as described above for qRT-PCR analysis. Flow cytometry data analysis was performed usingFlowJo v.10 software (BD Biosciences).

### In vitro *P. aeruginosa* infection and LPS treatment of FACS-sorted corneal and spleen Mϕ

A protocol described previously (22, 23) was followed with minor modifications. Briefly, FACS-sorted corneal ResMϕ and spleen Mϕ were plated in complete Mϕ media (DMEM/F12 + 10 mM l-glutamine [Life Technologies] +10% [v/v] FBS [HyClone] + M-CSF [2 ng/ml; R&D Systems]) in a 48-well plate and challenged with *P. aeruginosa* (strain 19660, multiplicity of infection [MOI] 5; ATCC) orLPS (100 ng/ml) (from *P. aeruginosa* serotype 10; Sigma-Aldrich). Six hours later, cells were harvested for miRNA-proof total RNA preparation using the miRVana miRNA Isolation Kit as described previously (22).

### Target luciferase reporter assays

Target luciferase reporter constructs of mouse Runx1 (MmiT072689a-MT06), which contains 3′ untranslated region (UTR) for mouse Runx1 (NM_001111023.2), with predicted target sites for miR-183 and miR-96, and mouse Maf (MmiT024436-MT06), which carries 3′ UTR of mouse Maf (NM_001025577.2), with predicted target sites for miR-182 and miR-96, downstream of a firefly luciferase cassette in the pEZX-MT06 vector, were purchased from GeneCopoeia. The pEZX-MT06 vector also carries a constitutively expressed Renilla luciferase cassette for transfection control (GeneCopoeia). Fifty nanograms of the construct plasmid DNA were cotransfected with miRNA mimics (miR-183 and/or miR-96 mimics for Runx1, miR-182, and/or miR-96 mimics for Maf or negative-control oligonucleotide duplex with scrambled sequences [scr]) (10 nM; Ambion/Thermo Fisher Scientific) or miRCURY LNA miRNA Power Inhibitors (anti-mmu-miR-183 and/or anti-mmu-miR-96 for Runx1, anti-mmu-miR-182 and/or anti-mmu-miR-96 for Maf, or negative-control oligonucleotides with scr) (10 nM; Exiqon/QIAGEN) into Raw264.7 cells (ATCC) using Lipofectamine 2000 (Invitrogen) as we described previously (28). Forty-eight hours later, we harvest the cells for dual luciferase assays using the Luc-Pair miR Luciferase Assay System (GeneCopoeia) and a GloMax Plate Reader (Promega, Madison, WI). Relative luciferase activity was calculated as firefly luciferase activity normalized by Renilla luciferase activity. All experiments were performed with five replicates under each condition (*n* = 5).

### Statistical analysis

When the comparison was made among more than two conditions, one-way ANOVA with Bonferroni multiple comparison test was employed (GraphPad Prism); adjusted *p <* 0.05 was considered significant. Otherwise, a two-tailed Student t test was used to determine the significance; *p* < 0.05 was considered significant. Each experiment was repeated at least twice to ensure reproducibility and data from a representative experiment are shown. Quantitative data are expressed as the mean ± SEM.

## RESULTS

### miR-183/96/182 regulates corneal innate IL-17 and IL-10 responses to *P. aeruginosa* infection

**FIGURE 1.**
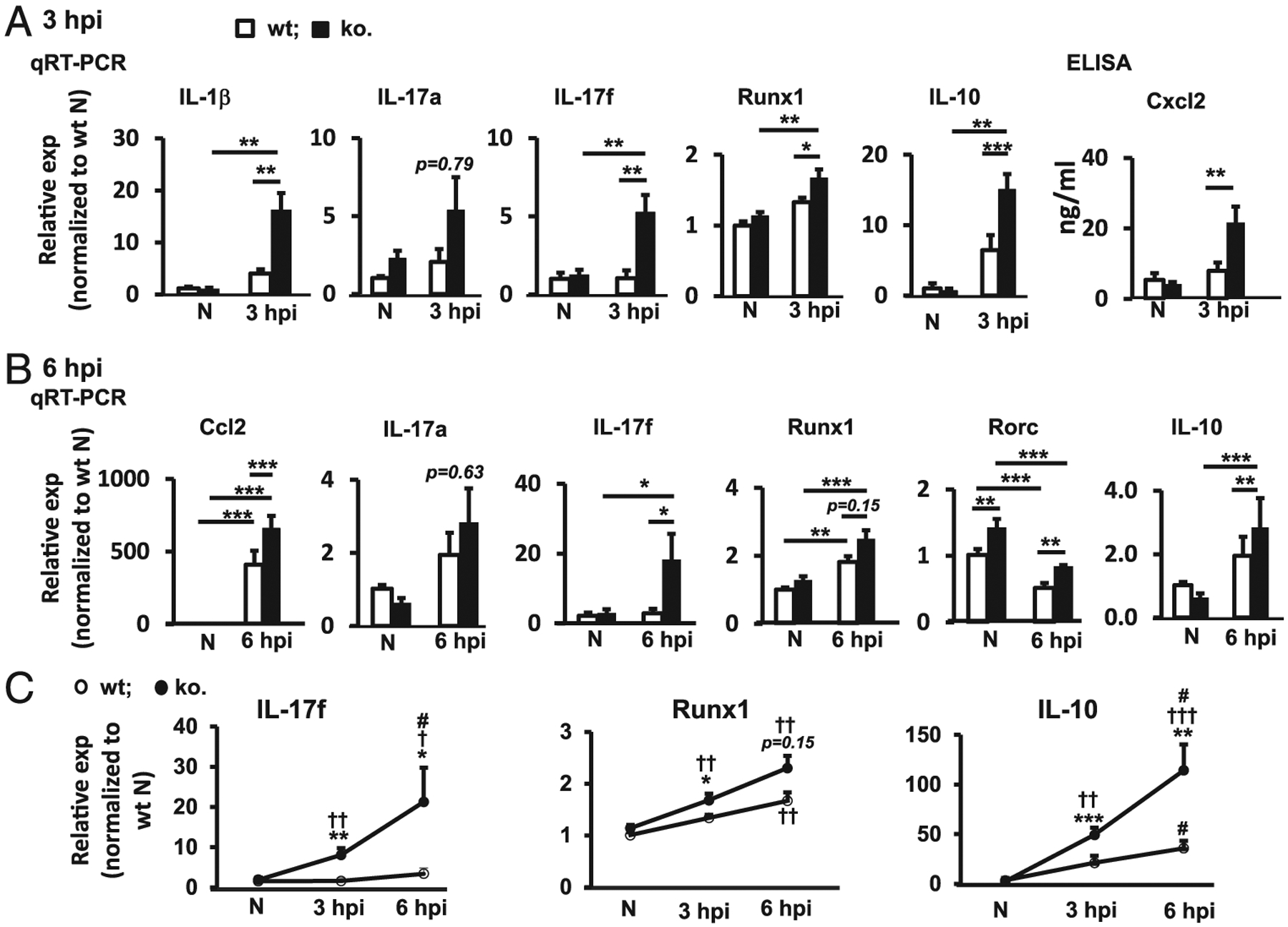
Inactivation of miR-183/96/182 resulted in increased cytokine production in the cornea at early-stage *P. aeruginosa* infection. (**A** and **B**) qRT-PCR and ELISA analyses of key cytokines and related transcription factors in the cornea at 3 (A) and 6 hpi (B). Relative expression (exp) is normalized to wt noninfected contralateral eye (N). *n* = 5 per group. *p < 0.05, **p < 0.01, ***p < 0.001. (**C**) Dynamic changes of IL-17f, Runx1, and IL-10 in the corneas of miR-183/96/182 ko and wt control mice at 3 and 6 hpi by qRT-PCR analysis. *p < 0.05, **p < 0.01, ***p < 0.001, comparison between wt versus ko; ^†^*p* < 0.05, ^††^*p* < 0.01, †††*p* < 0.001, comparison with the noninfected control; ^#^*p* < 0.05, comparison between 6 versus 3 hpi.

Upon acute inflammation, neutrophils are the first cells to accumulate in tissues (29). In cornea, infiltrating neutrophils are not recruited to the stroma until 3 hpi (30). Therefore, measurement of corneal cytokine levels within 3 hpi reflects the innate immune/inflammatory response of the cornea to *P. aeruginosa* infection. To determine whether miR-183/96/182 plays a role in this response, we performed *P. aeruginosa* infection (19660; ATCC) in young adult (8–12 wk old) miR-183/96/182 ko and age-matched wt control mice and harvested their corneas at 3 hpi for gene-expression analysis. qRT-PCR-based, low-density array screening and individual qRT-PCR confirmation assays demonstrated that proinflammatory cytokines, including IL-1β and IL-17f, were significantly upregulated in the infected cornea of ko versus wt controls (Fig. 1A). IL-17a showed a similar trend. Consistently, Runx1, a transcription factor known to play important roles in IL-17 production (31–33), was significantly upregulated (Fig. 1A). Intriguingly, the level of anti-inflammatory cytokine IL-10 also was elevated (Fig. 1A).

To evaluate whether these gene-expression changes in *P. aeruginosa*-infected cornea of miR-183/96/182 ko mice persist with time in the early stage of *P. aeruginosa* keratitis, we performed *P. aeruginosa* infection and harvested the cornea at 6 hpi. Consistent with the result at 3 hpi, both IL-17f and anti-inflammatory cytokine IL-10 were further upregulated at 6 hpi (Fig. 1B, 1C). Runx1 and another transcription factor, Rorc, which encodes the orphan nuclear receptor RORγt, a key lineage-specific transcription factor required for IL-17 production (34,35), were also significantly elevated in the cornea of miR-183/96/182 ko versus wt mice (Fig. 1B, 1C). These results suggest that miR-183/96/182 modulates the corneal innate IL-17 and IL-10 response to *P. aeruginosa* infection.

Compared with IL-17a, IL-17fshowed much higher expression levels in the *P. aeruginosa-infected* cornea in both ko and wt controls (Supplemental Fig. 1). At 3 hpi, the level of IL-17fwas ~22. 7- and 8.9-fold higher than IL-17a in wt and ko mice, respectively (Supplemental Fig. 1A, 1B); at 6 hpi, IL-17fwas ~4.6- and 16.3-fold higher than IL-17a in the wt controls and ko mice, respectively (Supplemental Fig. 1C, 1D).

Compared with wt control mice, the miR-183/96/182 ko mice showed a more rapid and robust IL-17 response to *P. aeruginosa* infection (Supplemental Fig. 1E, 1F). At 3 dpi, IL-17f was significantly upregulated (by ~4.3-fold) in ko mice, but not in the wt controls (Supplemental Fig. 1B, 1F). At 6 hpi, it was further elevated (by ~ 7.8-fold) in the ko mice, whereas only modestly upregulated (by ~3.8-fold) in the wt mice (Supplemental Fig. 1D, 1F). These data suggest that IL-17f plays a major role in the early corneal innate response to *P. aeruginosa* infection and that miR-183/96/182 regulates this response.

### miR-183/96/182 regulates the number of CRICs, including ResMϕ

**FIGURE 2.**
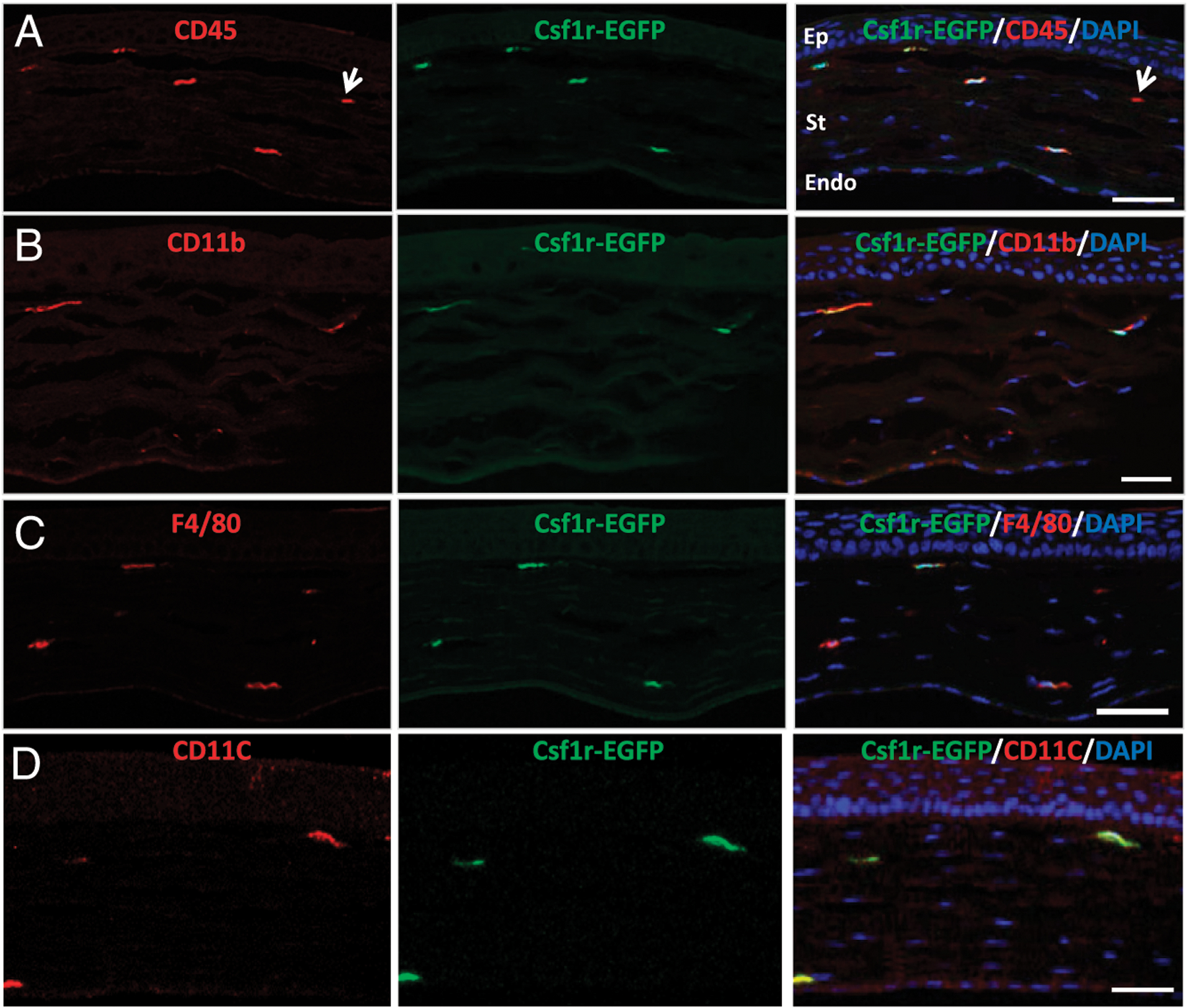
Csf1r-EGFP^+^ cells in the cornea are myeloid-derived MPS cells. Immunofluorescence of CD45 (**A**), CD11b (**B**), F4/80 (**C**), and CD11c (**D**) of cross-sections of the cornea of naive, young adult (8 wk old) Csf1r-EGFP(+);miR-183C^GT/+^ mice. The arrow in (A) points a CD45^+^EGFP^−^ cell. Scale bar, 50 μm. Endo, endothelial layer; Ep, epithelial layer; St, stromal layer.

**FIGURE 3.**
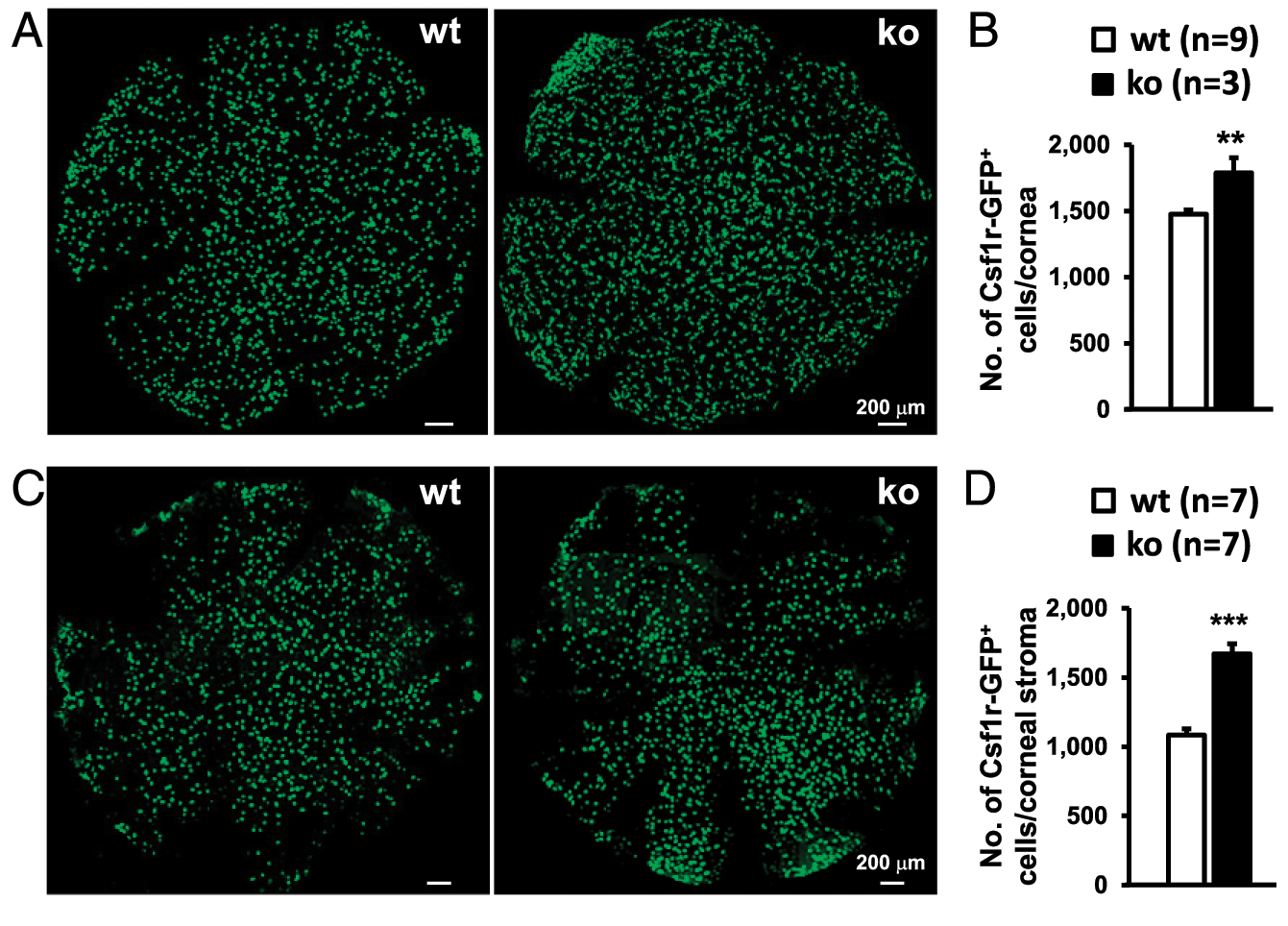
miR-183/96/182 ko mice have more Csf1r-EGFP^+^ CRICs. Representative confocal images of flatmounts of the whole cornea of naive P21 (**A**) or corneal stroma of 8–12-wk-old young adult (**C**) miR-183/96/182 ko and wt mice on the background of Csf1r-EGFP. (**B** and **D**) Comparison of the numbers of Csf1r-EGFP^+^ cell count per cornea between ko and wt mice. **p < 0.01, ***p < 0.001.

**FIGURE 4.**
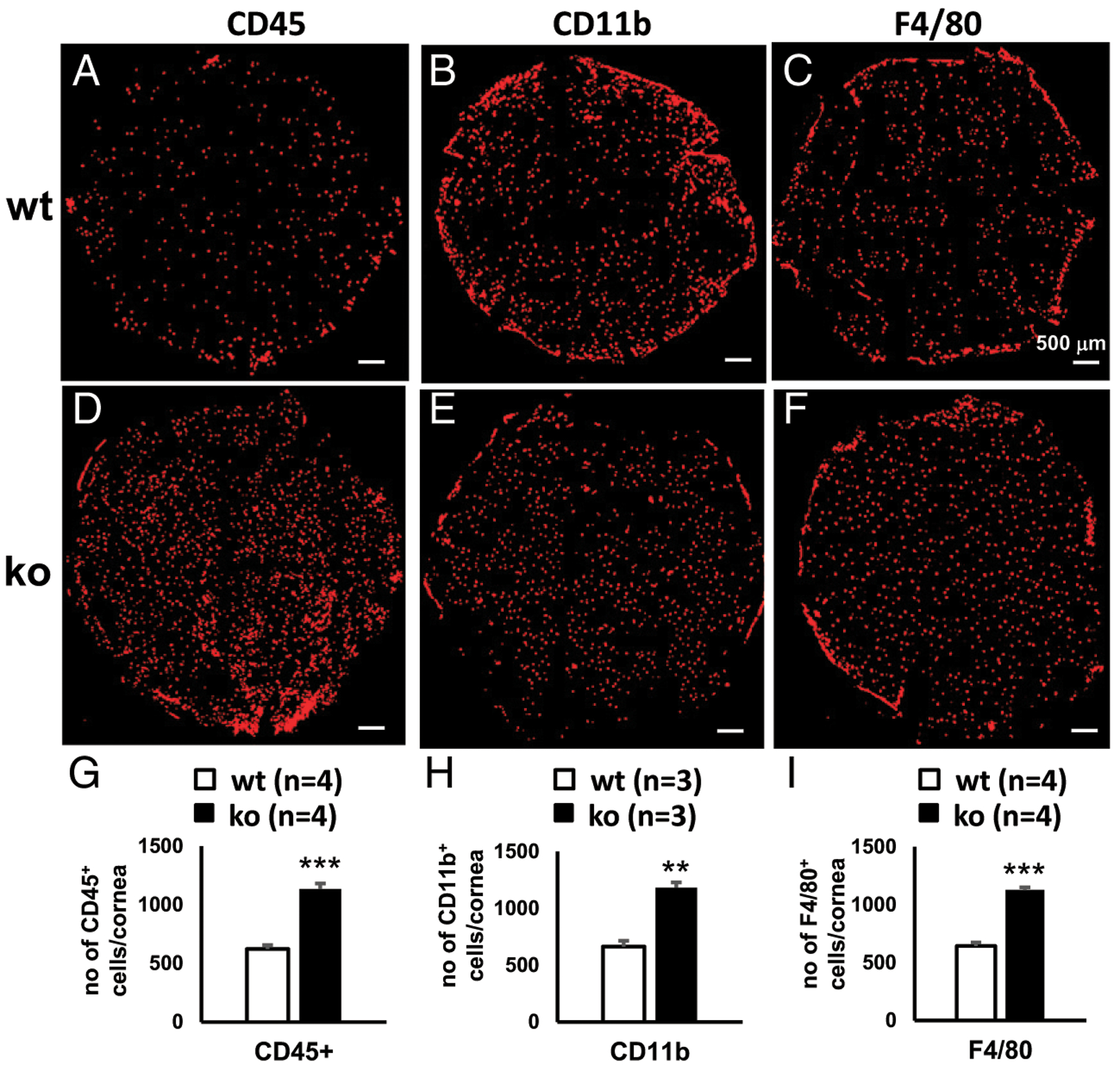
Inactivation of miR-183/96/182 resulted in increased number of CRICs. Immunofluorescence of CD45 (**A**, **D**, and **G**), CD11b (**B**, **E**, and **H**), and F4/80 (**C**, **F**, and **I**) of flatmount corneal stroma of wt (A–C) and miR-183/96/182 ko mice (D–F). (G–I) Comparison of the numbers per cornea of CD45^+^ (G), CD11b^+^ (H), and F4/80^+^ cells (I) in wt and ko mice. **p < 0.01, ***p < 0.001.

Csf1r (CD115, encoded by c-fms gene) is the receptor for M-CSF (M-CSF or Csf-1) and is required for the development, differentiation, proliferation, and survival of the myeloid-derived MCs, Mϕ, and DCs—the mononuclear phagocyte system (MPS) (2, 36). The Csflr-EGFP, also known as MacGreen, mouse, uses c-fms gene promoter plus enhancer to drive EGFP expression in the MPS cells (27,37). To directly study the roles of miR-183/96/182 in corneal resident innate immunity, we bred the miR-l83/96/l82 ko allele, miR-183C^GT^, into the MacGreen mice to produce miR-183/96/182 ko [Csf1r-EGFP(+);miR-183C^GT/GT^] and wt control mice [Csf1r-EGFP(+);miR-183C^+/+^] as well as heterozygous mice [Csf1r-EGFP(+);miR-183C^GT/+^]. Coimmunofluorescence of corneal cross-sections of naive, young adult Csf1r-EGFP(+);miR-183C^GT/+^ mice showed that all Csf1r-EGFP^+^ CRICs express the panleukocyte marker CD45 (Figs. 2A, 3, 4A, 4D, 4G). Most of these cells coexpress myeloid marker CD11b (Figs. 2B, 4B, 4E, 4H), Mϕ marker F4/80 (Figs. 2C, 4C, 4F, 4I), and DC marker CD11c (Fig. 2D), confirming that Csf1r-EGFP^+^ CRICs represent the resident MPS cells in the cornea.

To determine whether miR-183/96/182 regulates Csf1r-EGFP^+^ CRICs, we first performed confocal microscopy on whole-cornea flatmounts of naive, P21 miR-183/96/182 ko and age-matched wt control mice on the background of Csf1r-EGFP. On average, we detected 1475 ± 34 (SEM) EGFP^+^ MPS cells per cornea (*n* = 9) in the wt control mice (Fig. 3A, 3B). These cells were evenly distributed throughout the cornea in a tiled or a mosaic pattern (Fig. 3A), reminiscent of the microglial distribution in the parenchyma of the brain (38, 39) and the retina (40, 41). In the ko mice, the Csf1r-EGFP^+^ cells showed a similar distribution pattern (Fig. 3A); however, the number of Csf1r-EGFP^+^ cells in the cornea of ko mice (~1788 ± 115 per cornea, *n* = 3) was significantly increased (by ~21%) when compared with their wt controls (Fig. 3B).

To confirm this result in adult mice, we performed confocal microscopy on the flatmount corneal stroma of naive, young adult (8–12 wk old) ko and age-matched wt control mice. As in the P21 mice, Csf1r-EGFP^+^ cells showed similar distribution from the periphery to the center of the cornea (Fig. 3C); the number of Csf1r-EGFP^+^ cells in the corneal stroma of ko mice (~1672 ± 71 per cornea, *n* = 7) was significantly increased (by ~54%) compared with their age-matched wt controls (1084 ± 45/cornea, *n* = 7) (Fig. 3D). These data suggest that miR-183/96/182 has a significant regulatory role in the number of steady-state corneal resident MPS cells.

To further validate these results and to gain more insight into the roles of miR-183/96/182 in CRICs, we performed flatmount immunofluorescence on corneal stroma of naive mice with Abs against CD45, CD11, and F4/80. Similar to Csf1r-EGFP^+^ cells, CD45^+^, CD11b^+^, and F4/80^+^ CRICs were evenly distributed in the corneal stroma in both ko and wt control mice (Fig. 4A–F). The ko mice showed significantly increased numbers of CD45^+^ (Fig. 4A, 4D, 4G), CD11b^+^ (Fig. 4B, 4E, 4H), and F4/80^+^ cells (Fig. 4C, 4F, 4I, Table I). This result confirms that miR-183/96/182 regulates the number of steady-state CRICs, including corneal ResMϕ.

**FIGURE 5.**
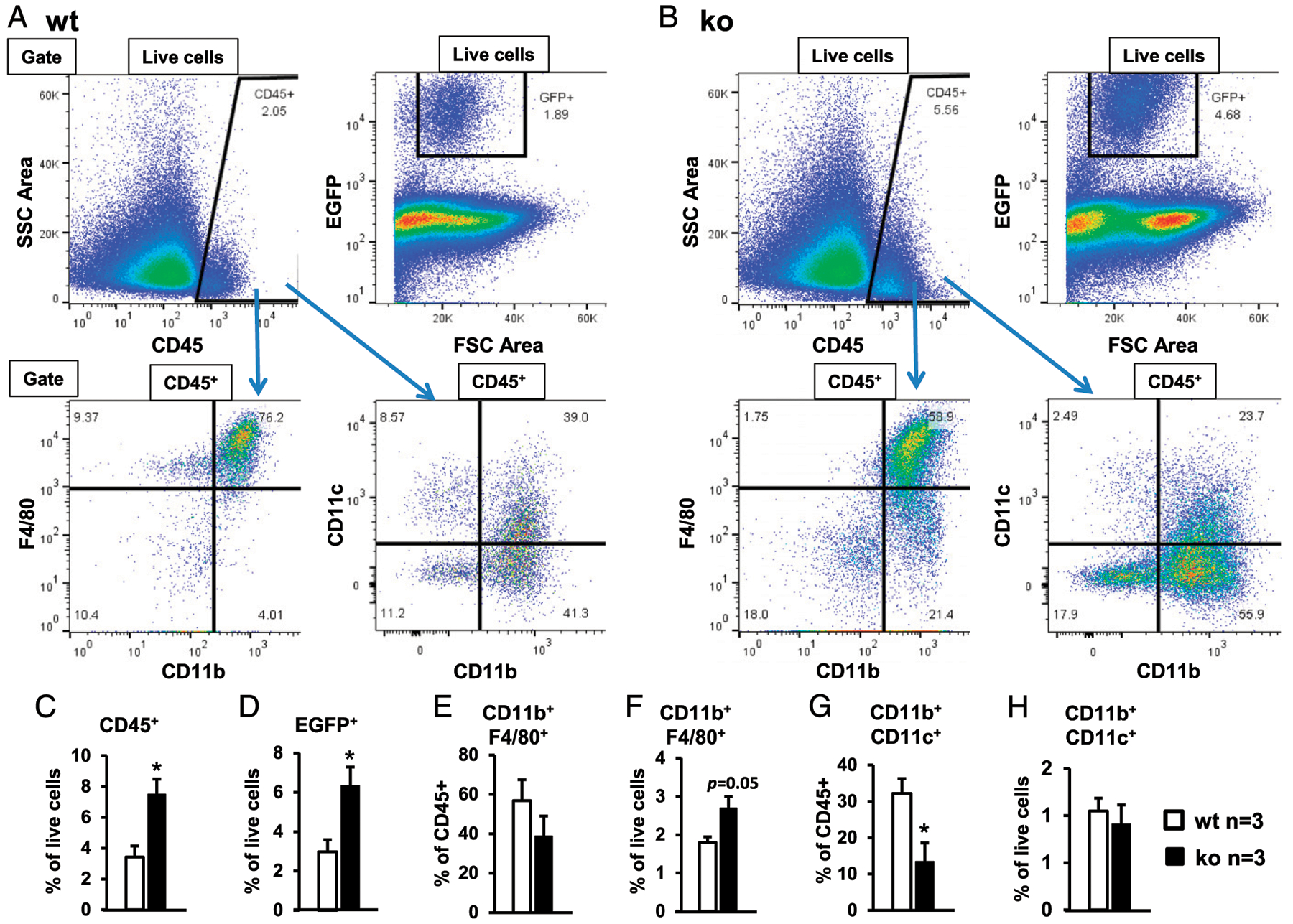
miR-183/96/182 modulates the number and composition of CRICs. Flow cytometry of corneal cells of naive, young adult (8–12 wk old) wt control (**A**) and miR-183/96/182 ko mice (**B**) on the background of Csf1r-EGFP. Corneal cells from five to nine naive mice of each genotype were pooled for each flow cytometry experiment. Data from three experiments were compiled in (**C**)–(**H**). (C) Percentage of CD45^+^ cells in total live cells. (D) Percentage of Csf1r-EGFP^+^ cells in total live cells. (E) Percentage of CD11b^+^F4/80^+^ cells in CD45^+^ cells. (F) Percentage of CD11b^+^F4/80^+^ cells in total live cells. (G) Percentage of CD11b^+^CD11c^+^ cells in CD45^+^ cells. (H) Percentage of CD11b^+^CD11c^+^ cells in total live cells. *p < 0.05.

To further confirm the flatmount microscopy findings, we performed flow cytometry on the corneal cells of naive, young adult (8–12 wk old) miR-183/96/182 ko and wt mice on the background of Csf1r-EGFP. Our result showed that 2.98 ± 0.62% oftotal live cells in wt controls (Fig. 5A, 5D), whereas 6.33 ± 0.96% in the ko mice (Fig. 5B, 5D), were Csf1r-EGFP^+^, confirming a significant increase of Csf1r-EGFP^+^ MPS cells in the cornea of ko versus wt mice. Consistently, 3.43 ± 0.71% of total live cells in wt controls (Fig. 5A, 5C), whereas 7.49 ± 1.00% in ko mice, were CD45^+^ (Fig. 5B, 5D), confirming a significantly increased number of total CRICs in the cornea of ko mice. As predicted, the vast majority of Csf1r-EGFP^+^ cells (>90%) were CD45^+^ in both wt and ko mice (Supplemental Fig. 2). Although the percentage of CD11b^+^F4/80^+^ ResMϕ in the CD45^+^ cells in the ko mice showed no significant change compared with the wt controls (Fig. 5E), CD11b^+^F4/80^+^ ResMϕ in total live cells was increased in the ko (2.69 ± 0.30%) versus wt control cornea (1.89 ± 0.15%. *p* = 0.05) (Fig. 5F) because ko mice had an increased percentage of CD45^+^ cells (Fig. 5C). Intriguingly, the percentage of CD11b^+^CD11c^+^ DCs among CD45^+^ cells appeared to be decreased in the ko (13.46 ± 5.12%) versus wt controls (32.20 ± 4.10%) (Fig. 5G); however, the total number of CD11b^+^CD11c^+^ cells among all live cells showed no significant change between ko and wt control mice (Fig. 5H) because of increased CD45^+^ cells in the ko mice (Fig. 5C). Collectively, these data confirm that miR-183/96/182 regulates the number as well as the composition of the CRICs; inactivation of miR-183/96/182 results in increased number of CRICs, including corneal ResMϕ.

### *P. aeruginosa* infection and LPS treatment induce miR-183/96/182 in corneal ResMϕ, which in turn negatively regulates the expression of the LPSR, TLR (TLR4)

**FIGURE 6.**
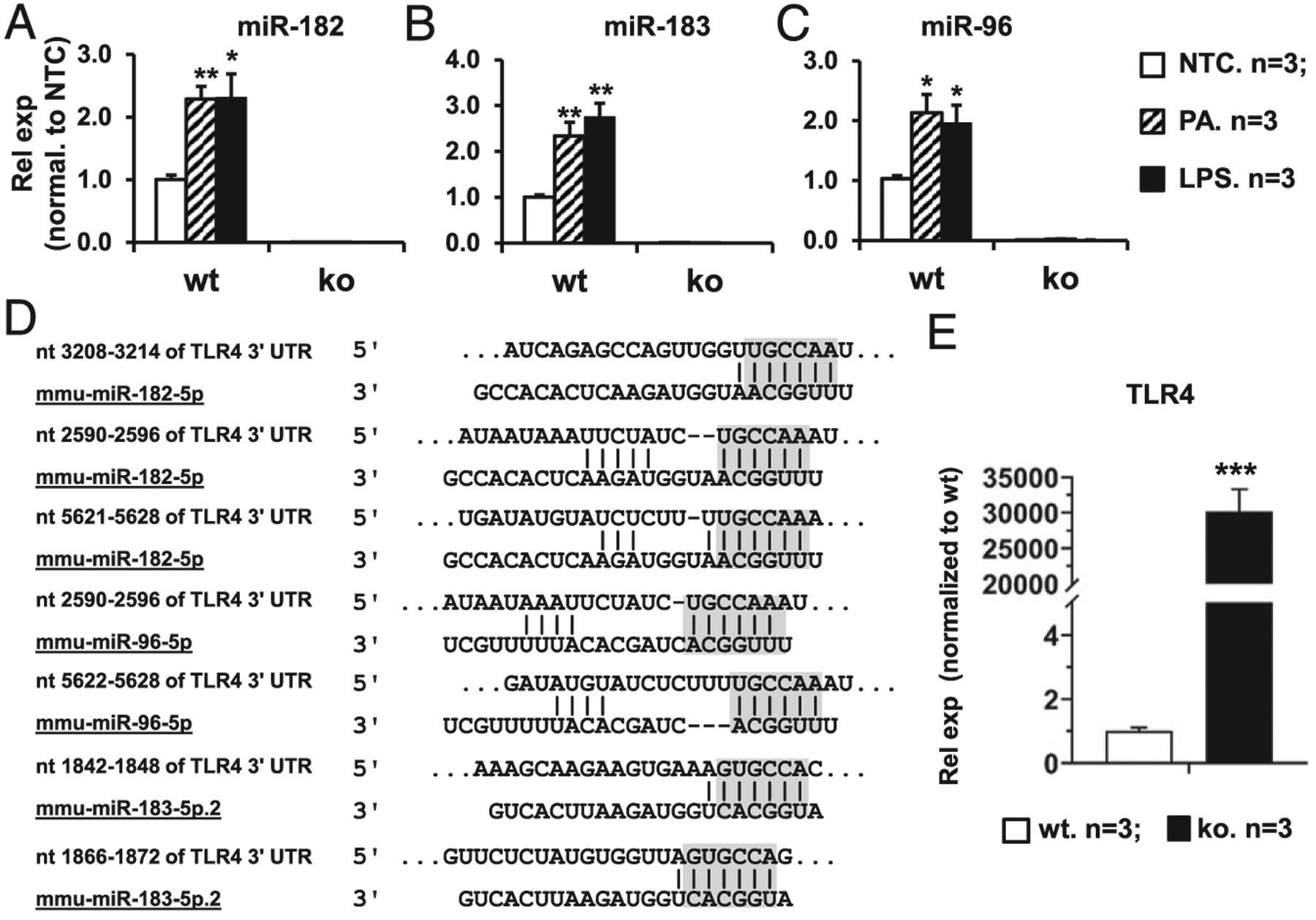
*P. aeruginosa* and LPS induce the expression of miR-183/96/182 in corneal ResMϕ, which in turn negatively regulate LPS receptor, TLR4. (**A–C**) qRT-PCR analysis of miR-183/96/182 expression in corneal ResMϕ in response to a 6-h treatment of *P. aeruginosa* (MOI = 5) or LPS (100 ng/ml). Relative expression levels (Rel exp) were normalized to no-treatment control (NTC). *n* = 3 per group. *p < 0.05, **p < 0.01. (**D**) Sequencing alignment of miR-183, miR-96, or miR-182 with their predicted target sites in the 3′ UTR of mouse TLR4. Gray-shaded residues represent the seed sequences of miRNAs and corresponding predicted target sequences. (**E**) qRT-PCR of TLR4 in FACS-sorted corneal ResMϕ (CD45^+^CD11b^+^F4/80^+^). Rel exp is normalized to wt control mice. ***p < 0.001.

Previously, we showed that *P. aeruginosa* and LPS treatment induced the expression of miR-183/96/182 in Raw264.7 Mϕ-like cells (23). To test this in corneal ResMϕ, FACS-sorted corneal ResMϕ (CD45^+^CD11b^+^F4/80^+^), as well as spleen Mϕ as a control, were challenged with *P. aeruginosa* (19660, MOI5; ATCC) and LPS (100 ng/ml) for 6 h. qRT-PCR assays showed that, similar to our previous reports in peritoneal Mϕ (22) and Raw264.7 cells (23), in nontreated corneal ResMϕ, miR-182 has the highest expression among the three miRNAs of the cluster [~2.1-fold higher than miR-183, and ~ 12-fold higher than miR-96 (Supplemental Fig. 3A)]. The relative expression levels of these miRNAs showed a similar ratio in the spleen Mϕ (Supplemental Fig. 3B), suggesting a similar posttranscriptional maturation mechanism for the three miRNAs of the cluster in Mϕ of different tissue sources. Intriguingly, the overall expression levels of miR-183, miR-182, and miR-96 in corneal ResMϕ are significantly lower (~410-, 1176-, and 964-fold, respectively) than their levels in spleen Mϕ (Supplemental Fig. 3A, 3B), suggesting a possible tissue-specific transcriptional regulation on miR183/96/182 expression in corneal ResMϕ and spleen Mϕ. More importantly, *P. aeruginosa* and LPS treatment resulted in a significant upregulation of miR-182, miR-183, and miR-96 in the corneal ResMϕ (Fig. 6A–C) as well as in spleen Mϕ (Supplemental Fig. 3F–H), suggesting that the expression of miR-183/96/182 is responsive to *P. aeruginosa* and LPS treatment. LPS is a major component of the outer membrane of *P. aeruginosa* and a virulence factor that causes inflammatory responses through activation of TLR4 and its cofactors (42, 43). *P. aeruginosa* and LPS treatment resulted in a similar extent of induction of miR-183/96/182 (Fig. 6A–C), suggesting that P. aeruginosa-induced miR-183/96/182 upregulation in Mϕ is mediated by LPS/TLR4 pathway and that miR-183/96/182 may mediate *P. aeruginosa*- and LPS-induced immune/inflammatory response of the Mϕ.

Recently, several groups reported that TLR4 is a direct target of miR-182 by target luciferase reporter assays (44–49). Consistent with these reports, our target prediction analysis using the TargetScan algorithm (TargetScan.org) showed multiple putative target sites of miR-182, miR-96, and miR-183 in the 3′ UTR of mouse TLR4 transcript (Fig. 6D). If it is a direct target of miR-183/96/182 in corneal ResMϕ, we expect that TLR4 would be upregulated in corneal ResMϕ of naive miR-183/96/182 ko versus wt control mice. To test this hypothesis, we performed qRT-PCR analysis on FACS-sorted corneal ResMϕ. As we predicted, the result showed that TLR4 is indeed significantly increased in the corneal ResMϕ of the miR-183/96/182 ko mice versus the wt controls (Fig. 6E), suggesting TLR4 is targeted by the cluster in corneal ResMϕ. Collectively, our data suggest a negative feedback regulation of miR-183/96/96 on the TLR4 signaling pathway.

### miR-183/96/182 modulates IL-17f and IL-10 expression in corneal ResMϕ through targeting Runx1 and Maf, respectively

**FIGURE 7.**
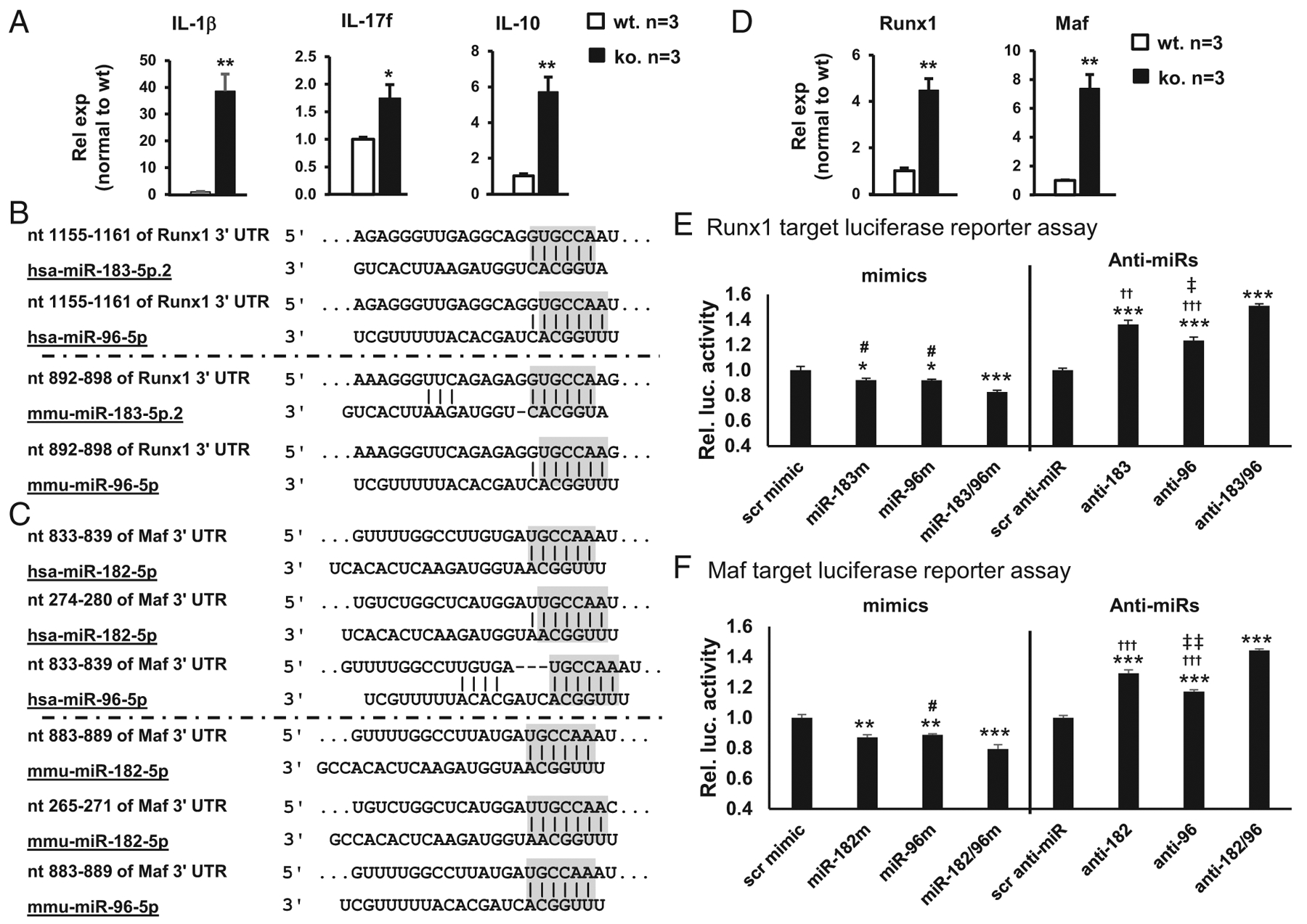
miR-183/96/182 regulates IL-17 and IL-10 production in corneal ResMϕ through targeting Runxi and Maf, respectively. (**A**) qRT-PCR of IL-1β, IL-17f, and IL-10 in FACS-sorted corneal ResMϕ (CD45^+^CD11b^+^F4/80^+^). Relative expression (Rel exp) is normalized to wt control mice. (**B**–**F**) Runx1 and Maf are targeted by miR-183/96/182. (B and C) Sequencing alignment of miR-183, miR-96, or miR-182 with their target sites in the 3′ UTR of human and mouse Runx1 (B) or Maf transcripts (C). Gray-shaded residues represent the seed sequences of miRNAs and corresponding predicted target sequences. (D) qRT-PCR analysis on Runx1 and Maf in FACS-sorted corneal ResMϕ. *n* = 3 per genotype. (E and F) Target luciferase reporter assay of mouse Runx1 and Maf, respectively. *n* = 5 for each condition. *p < 0.05, **p < 0.01, ***p < 0.001 in comparison with negative controls with scr. ^#^*p* < 0.05 in comparison with the ones simultaneously transfected with miR-183/96 mimics (miR-183/96m) or miR-182/96 mimics (miR-182/96m). ^††^*p* < 0.01, ^†††^*p* < 0.001 in comparison with the ones simultaneously transfected with anti-miR-183/96 (anti-183/96) or anti-miR-182/96 (anti-182/96). ^‡^*p* < 0.05, ^‡‡^*p <* 0.01 in comparison with ones transfected with anti-miR-183 (anti-183) or anti-miR-182 (anti-182). Rel. luc.activity, relative luciferase activity.

To test whether miR-183/96/182 regulates the expression of key immune/inflammatory genes in corneal ResMϕ, we performed qRT-PCR in FACS-purified CD45^+^CD11b^+^F4/80^+^ cells from naive miR-183/96/182 ko and age-matched wt control mice. Our results showed that corneal ResMϕ from ko mice had significantly increased level of IL-1β, IL-17f, and anti-inflammatory IL-10 (Fig. 7A), whereas no significant difference was detected in the CD45^−^ nonhematopoietic cells of the cornea (Supplemental Fig. 4B), suggesting that corneal ResMϕ are innate IL-17- and IL-10-producing cells and that miR-183/96/182 limits the expression of key cytokines, including IL-1β (an M1 marker) and IL-10 (an M2 marker) as well as IL-17f in corneal ResMϕ. This pattern is reminiscent of the cytokine expression profile in the early corneal immune/inflammatory response to *P. aeruginosa* infection at 3 and 6 hpi (Fig. 1), suggesting that miR-183/96/182’s regulation on ResMϕ contributes to its overall function in corneal innate immune response at an early stage of P. *aeruginosa* infection.

In an effort to understand the underlying mechanisms, we performed target prediction using the TargetScan algorithm (TargetScan.org). We identified that Runx1, which is a key transcription factor important for IL-17 production (31–33) and significantly upregulated in the *P. aeruginosa-infected* cornea of ko versus wt control mice (Fig. 1), is a predicted target ofmiR-183 and miR-96 in both mouse and human (Fig. 7B); whereas Maf (avian musculoaponeurotic fibrosarcoma oncogene homolog), a transcription factor critical for IL-10 production (50), is a predicted target of miR-182 and miR-96 with multiple predicted target sites in both mouse and human (Fig. 7C). If miR-183/96/182 targets Runx1 and Maf in corneal ResMϕ in vivo, we expect that expression of Runx1 and Maf is upregulated in miR-183/96/182 ko mice. To determine whether this is the case, we performed qRT-PCR in FACS-purified corneal ResMϕ (CD45^+^CD11b^+^F4/80^+^). As expected, both Runx1 and Maf were significantly upregulated in the corneal ResMϕ of ko versus wt control mice (Fig. 7D), suggesting that miR-183/96/182 targets Runx1 and Maf in vivo.

To further confirm that Runx1 and Maf are direct targets of miR-183/96/182, we performed target luciferase reporter assays in Mf-like Raw264.7 cells. Our result showed that cotransfection of luciferase reporter constructs with miR-183 and/or miR-96 mimics (for Runx1) or miR-182 and/or miR-96 (for Maf) resulted in significantly reduced luciferase activity, suggesting Runx1 and Maf are directly targeted by miR-183/96 and miR-182/96, respectively (Fig. 7E, 7F). Interestingly, cotransfection of both miR-183/96 (for Runxi) or miR-182/96 mimics (for Maf) led to additional significant decrease of the luciferase activity of both Runx1 and Maf constructs, compared with cotransfection with any of the miRNA mimic singularly, suggesting a modest additive effect of miR-183/96 on Runx1 and miR-182/96 on Maf regulations (Fig. 7E, 7F). Consistently, cotransfection of anti-miR-183/96 (for Runx1) and anti-miR-182/96 (for Maf) resulted in significantly increased luciferase activities (Fig. 7E, 7F). miR-183/96/182 is endogenously expressed in Raw264.7 cells (22,23). In combination with our current data, it suggests that downregulation of miR-183/98 (for Runx1) or miR-183/96 (for Maf) disinhibited the endogenous miR-183/96/182’s negative regulation on the expression of Runx1 and Maf, respectively. Consistent with that miR-96 is expressed at a much lower level than miR-183 and miR-182 in Raw264.7 cells (23), anti-miR-96-induced luciferase activity is more modest than the ones caused by anti-miR-183 (for Runx1) or anti-miR-182 (for Maf) (Fig. 7E, 7F). Despite its low-level expression and modest regulatory effect, the fact that anti-miR-96 resulted in a significant disinhibition of the luciferase activity in both Runx1 and Maf target luciferase reporter constructs argues that endogenous miR-96 have a significant function regulating the expression of Runx1 and Maf. Furthermore, cotransfection of both anti-miR-183/96 (for Runx1) or both anti-miR-182/96 (for Maf) resulted additional increase of the luciferase activity, confirming an endogenous additive effect on miR-183/96 onRunx1 and miR-182/96 on MAF in Raw264.7 cells. Collectively, our data suggest that Runx1 and Maf are direct targets of miR-183/96/182 in ResMϕ; upregulation of Runx1 and Maf may have contributed to the increased expression of IL-17f and IL-10 observed in the ResMϕ (Fig. 7A), as well as in the early corneal response to *P. aeruginosa* infection of the ko mice (Fig. 1).

### miR-183/96/182 regulates the dynamic changes of corneal Csf1r-EGFP^+^ MPS cells in response to *P. aeruginosa* infection

**FIGURE 8.**
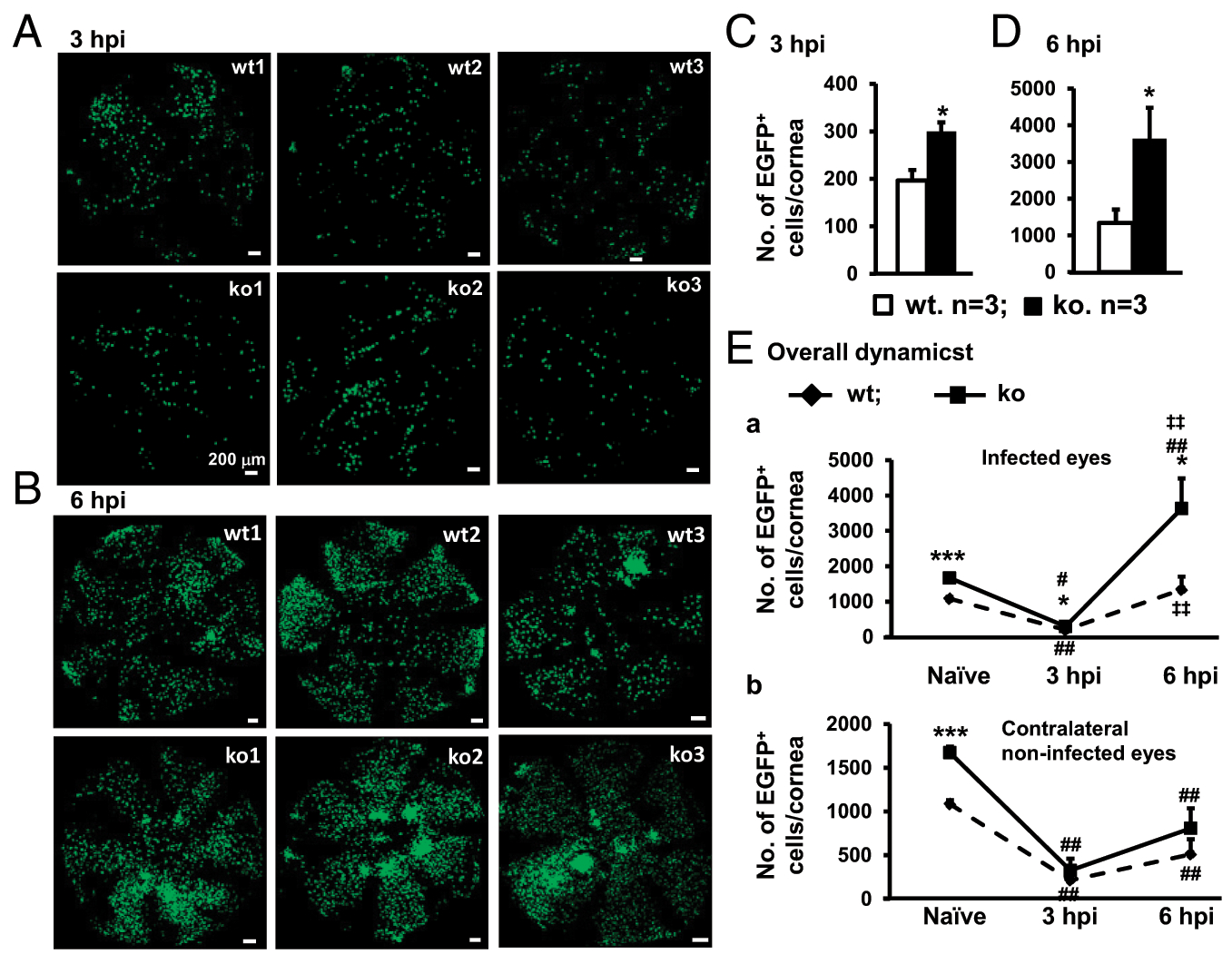
Inactivation of miR-183/96/182 changed the migratory dynamics of Csf1r-EGFP^+^ MPS cells upon *P. aeruginosa* infection. Corneal stromal flatmount of young adult (8–12 wk old) miR-183/96/182 ko and age-matched wt contro(*n* = 3 per genotype) at 3 (**A** and **C**) and 6 hpi (**B** and **D**) in comparison with naive mouse cornea (*n* = 7 per genotype) (**E**). *p < 0.05, ***p < 0.001 in comparison of ko versus wt. ^#^*p* < 0.05, ^##^*p* < 0.01 in comparison with infected (E**a**) or contralateral eyes (E**b**) of *P. aeruginosa-infected* mice versus the ones of naive mice. ^‡‡^*p* < 0.01 in comparison with the numbers at 3 versus 6 hpi in ko (above the curves) and wt mice (below the curves).

Upon tissue damage and microbial infection, the dynamic interplay between ResMϕ and MC-derived infiltrating Mϕ plays important roles in the wound healing and the resolution of microbial infection (51–53). The so-called Mϕ disappearance reaction (51) is considered to allow the immune-regulatory ResMϕ to temporarily give way to proinflammatory ResMϕ and infiltrating Mϕ to evoke an acute inflammatory response before they reappear to contain the inflammation and promote tissue repair (51–53). Given that our data show that miR-183/96/182 has a regulatory role in the number of CRICs and gene expression of corneal ResMϕ, we hypothesized that miR-183/96/182 may modulate the dynamics of ResMϕ in response to *P. aeruginosa* infection. To test this hypothesis and directly observe the dynamic changes of CRICs in response to *P. aeruginosa* infection, we performed *P. aeruginosa* (19660; ATCC) infection in the cornea of ko and wt control mice on the Csf1r-EGFP background and harvested them at 3 and 6 hpi for confocal microscopy. Our results showed that, by 3 hpi, the numbers of Csf1r-EGFP^+^ CRICs in the corneal stroma of the infected eye were significantly decreased (by ~82%) in both ko and wt mice (Fig. 8A, 8C) compared with their corresponding naive controls (Fig. 3C, 3D), although the absolute number of Csf1r-EGFP^+^ CRICs in ko (300 ± 19 per cornea, *n* = 3) was still significantly higher than the wt controls (197 ± 22 per cornea, *n* = 3) (Fig. 8A, 8C, 8Ea, Supplemental Fig. 5, Table II). Intriguingly, the numbers of Csf1r-EGFP^+^ CRICs in the non-infected contralateral eyes were simultaneously decreased to a similar extent as the infected eyes in both ko and wt control mice (Fig. 8Eb, Supplemental Figs. 5, 6A, 6B, Table II). By 6 hpi, the number of Csf1r-EGFP^+^ MPS cells in infected cornea of wt control mice (1339 ± 367 per cornea, *n* = 3) returned to a level similar to that seen in the cornea of naive wt mice (1084 ± 45 per cornea, *n* = 7), whereas the number of Csf1r-EGFP^+^ MPS cells in the infected eyes of ko mice was recovered to a significantly higher level when compared with either the infected cornea of wt controls or the naive ko cornea (Fig 8B, 8D, 8E, Supplemental Fig 5, Table II). In fact, the number of Csf1r-EGFP^+^ cells in the infected ko cornea at 6 hpi (3633 ± 848 per cornea, *n* = 3) was more than two times the level in naive ko cornea (1672 ± 71 per cornea, *n* = 7) (Fig. 8D, 8E), whereas the contralateral cornea of ko mice did not show a similar extent of increase (Fig. 8E, Supplemental Figs. 5, 6, Table II). Although the numbers of Csf1r-EGFP^+^ MPS cells of the contralateral eyes were moderately increased at 6 versus 3 hpi in both ko and wt control mice, they were significantly lower than the levels of their naive counterparts (~48 and 47% of ko and wt naive controls, respectively) (Fig. 8E, Supplemental Fig. 5B, 5D, 5F, Table II). These results suggest that miR-183/96/182 regulates not only the number of steady-state CRICs but also the dynamics of Csf1r-EGFP^+^ MPS (27, 37) upon bacterial infection, which may contribute to its overall impact on the development and resolution of *P. aeruginosa* keratitis (22).

## DISCUSSION

Previously, we showed that miR-183/96/182 promotes Th17 pathogenicity by negatively regulating transcription factor Foxo1 (24). In this study, we provide evidence that miR-183/96/182 also regulates innate IL-17 production in the cornea. We showed that corneal ResMϕ constitutively produce both IL-17f and IL-10; miR-183/96/182 regulates their expression by targeting key transcription factors for their production, Runx1 (31–33) and Maf (50, 54), respectively. Inactivation of miR-183/96/182 resulted in simultaneous upregulation of both IL-17f and IL-10 in ResMϕ, suggesting that, physiologically, miR-183/96/182 limits the production of innate IL-17f and IL-10 in corneal ResMϕ, contributing to the immune surveillance of the cornea under normal circumstance. The cornea is the interface between the eye and the external environment and is the start of the vision. It provides transparency and two thirds of the total refractive power of the eye for a clear vision (55, 56). A myriad of anatomical, physiological, and immunological features of the ocular surface work in concert to endow the eye with immune privilege and limit excessive inflammation in the cornea to fulfill its normal functions (4, 55–57). IL-17f is a potent inducer of proinflammatory cytokines/chemokines to promote granulopoiesis and neutrophil recruitment in various tissues (58–60). Limiting the production of IL-17f by miR-183/96/182 under physiological conditions may help prevent unnecessary inflammation and contribute to homeostasis of the cornea and ocular immune privilege. In regard to IL-10, it is mostly recognized as an anti-inflammatory cytokine; however, overproduction of IL-10 may suppress protective immune response and promote persistence and disseminate of bacteria and viruses within the host (61,62). Under physiological circumstances with little inflammation to suppress, miR-183/96/182 helps keep IL-10 low to prevent its inhibition on the immune surveillance function of corneal ResMϕ and other resident immune cells. However, in case of bacterial infection, these mechanisms that are beneficial under physiological conditions may limit the ability of the corneal ResMϕ to initiate a swift and sufficient innate immune/inflammatory response to bacterial infection. Inactivation of miR-183/96/182 releases its inhibition on IL-17f and IL-10 expression to enhance phagocytosis (63–66) and bacterial clearance capacity (58–60, 65, 66) of corneal ResMϕ and allow corneal ResMϕ to orchestrate an enhanced antibacterial inflammation and simultaneously help protect the integrity of the cornea and keeping the inflammatory response in check in early stages of *P. aeruginosa* infection. These effects may have contributed to the overall decreased severity of P. *aeruginosa* keratitis in the ko mice that we reported previously (22).

Our data suggest that that innate IL-17f plays a major role in the early corneal innate response to *P. aeruginosa* infection, based on its predominant expression levels in corneal ResMϕ of naive mice and at 3 and 6 hpi of *P. aeruginosa* infection, when compared with IL-17a. Unlike IL-17a, which is important for both T cell-dependent autoimmune and allergic responses and host defense response to microbial infection, IL-17f is specialized in host defense against mucoepithelial bacterial infection (58, 59). In this aspect, like IL-17a, IL-17f enhances host defense against bacterial infection by upregulation of proinflammatory cytokines and chemokines to promote granulopoiesis and neutrophil recruitment, Mϕ activation to boost their bactericidal activity through increased production of reactive oxygen species, and the production of antimicrobial peptides (58–60). The increased expression of IL-17fin the cornea and corneal ResMϕ in miR-183/96/182 ko mice may have contributed to the elevated levels of chemokines, IL-1β, Cxcl2, and Ccl2 in the cornea in early stages (3 and 6 hpi) of *P. aeruginosa* infection, which enhances the recruitment of infiltrating neutrophils and Mϕ, consistent with our observation of significantly increased Csf1r-EGFP^+^ MPS cells at 6 hpi of *P. aeruginosa* infection, suggesting that increased expression of IL-17f in the corneal ResMϕ has significant functional consequences. Because Mϕ express both receptors for IL-17f, IL-17RA and IL-17RC (58) as well as IL-10R (67), our data support an autocrine model for IL-17f and IL-10 in corneal ResMϕ—increased production of IL-17f and IL-10 by the corneal ResMϕ of miR-183/96/182 ko mice promotes the activation of its own receptors and downstream pathways to enhance the antibacterial defensive functions of corneal ResMϕ of miR-183/96/182 ko mice. This hypothesis appears to be consistent with our previous observation that inactivation of miR-183/96/182 results in enhanced production of reactive oxygen species/reactive nitrogen species and phagocytosis and bacterial killing capacity of Mϕ and decreased severity of *P. aeruginosa* keratitis that we described previously (22, 23). Further studies are needed to test this hypothesis.

It has been shown that other types of resident immune cells in the cornea (e.g., resident γδT cells within the epithelial layer also express IL-17) (8) and play important roles in acute inflammation, corneal wound healing and nerve regeneration (8, 9), herpes stromal keratitis (68), spontaneous autoimmune keratitis (69), ocular immune privilege, and corneal graft survival (15). Although our data demonstrated that corneal ResMϕ are innate IL-17- and IL-10-producing cells and are regulated by miR-183/96/182, we do not exclude the possibility that other CRICs also produce IL-17 and IL-10 in this context and that miR-183/96/182 may regulate these cells in the cornea. Whether miR-183/96/182 play a role in IL-17 production in γδT cells and other IL-17-producing cells remains to be determined.

Previously, we showed that in Mϕ-like Raw264.7 cells (70, 71) and peritoneal Mϕ (22, 23) as well as in Th17 cells (24), miR-183/96/182 promotes the production of proinflammatory cytokines by targeting DAP12 in Raw264.7 cells and peritoneal Mϕ (23) and transcription factor Foxo1 in Th17 cells (24); inactivation of miR-183/96/182 resulted in decreased production of proinflammatory cytokine in Raw264.7 and peritoneal Mϕ and IL-17 production in Th17 cells (22–24). This appears to be contradictory to our current report of an increased basal level of IL-17f in corneal ResMϕ of miR-183/96/182 ko mice through its regulation on Runx1, which resulted in an increased production of proinflammatory cytokines in the cornea at early stages (3 and 6 hpi) of *P. aeruginosa* infection. This discrepancy exemplifies the cell type specificity of the functions of miR-183/96/182 through targeting different downstream genes and signaling pathways. Numerous reports have shown that tissue-specific niches of ResMϕ imprint their tissue-specific identity and function (2, 72); specific niche in the cornea may have conferred corneal specific functions to the corneal ResMϕ to meet the specific functional demand of the cornea as discussed above.

In the current report, our data also showed that *P. aeruginosa* and LPS induce miR-183/96/182 expression through TLR4 and its cofactors (42, 43); in turn, miR-183/96/182 targets TLR4 and negatively regulates its expression, suggesting a negative feedback regulation of miR-183/96/182 on TRL4 signaling pathway. Inactivation of miR-183/96/182 results in significantly increased expression of TLR4 in the corneal ResMϕ of the ko mice, enhancing their response to *P. aeruginosa* and may have contributed to increased production of proinflammatory cytokines in the cornea at the early stages of *P. aeruginosa* infection.

miRNAs regulate their downstream genes by base pairing with their transcripts and inducing mRNA breakdown and/or translation inhibition. The major determinant of a target site is a short stretch of residues in the 3′ UTR of the transcript complementary to the so-called seed sequence nt 2–7 of a miRNA (18). Because such 6-mers are present in, on average, every ~ 4 kb, one miRNA is predicted to regulate up to hundreds of downstream target genes. However, recent studies further demonstrate that miRNAs function in a highly cell type-specific fashion based on the different transcriptomic contexts of different cell types. In each cell type, one miRNA targets small group(s) of genes, which are often involved in the same or related pathways or networks (23, 73–77). miRNAs are quantitative regulators of gene expression. Although the regulation on each individual target gene is often modest, the simultaneous, subtle regulations of multiple genes in concert impose significant functional consequence on a signaling pathway or network (73–76). In this study, we showed that miR-183/96/182 modulates three important pathways in corneal ResMϕ-TLR4 signaling pathway and innate IL-17f and IL-10 production through targeting three key genes, TLR4, Runx1, and Maf, respectively. Collectively, modulation on these pathways imposes significant functional regulation on corneal innate immunity. We predict that miR-183/96/182 may target other genes involved in other signaling pathways of the innate immunity. A genome-wide interactome study (75–77) on miR-183/96/182 and its functional target genes in corneal ResMϕ in comparison with peritoneal Mϕ, Th17 cells, and other cell types in which miR-183/96/182 also plays important roles (e.g., various sensory neurons) (20) will further elucidate the mechanism on how miR-183/96/182 imposes a global regulation on the tissue-specific functions of corneal ResMϕ and other cell types.

In addition to its role in the cytokine production function of corneal ResMϕ, our data suggest that miR-183/96/182 regulates the number of resident immune cells in the cornea, including ResMϕ. By a combined approach of immunofluorescence and confocal microcopy, we demonstrated that in naive mice, corneal ResMϕ are regularly distributed throughout the cornea in a tiled or a mosaic pattern reminiscent of the microglial distribution in the parenchyma of the brain (38,39) and the retina (40,41), suggesting a similar surveillance junction to constantly scan the microenvironment and detect the microbial invasion and other insults to the cornea. Intriguingly, naive miR-183/96/182 ko mice have significantly increased number of corneal ResMϕ, as well as total number of resident immune cells, suggesting that miR-183/96/182 regulates the number of steady-state CRICs, including ResMϕ. Therefore, our data support that miR-183/96/182 has a major regulatory role in the homeostasis and maintenance and innate immune/inflammatory response of the cornea to microbial infection through its regulation on both the functions of individual ResMϕ as well as the number of ResMϕ.

Corneal ResMϕ are considered to have at least two major sources: one population is from yolk sac-derived *erythro-myeloid* precursors. They took residence in the cornea as early as embryonic day 12.5 and share similar characteristics as the yolk sac-derived microglia in the brain; the other population is derived from definitive hematopoietic stem cells of the developing fetal liver and bone marrow and do not appear in the cornea until embryonic day 17.5 (53). Because Csf1r-EGFP labels both populations (27, 37), our current data could not distinguish whether the increased number of corneal ResMϕ in the miR-183/96/182 ko mice is a result of increased initial seeding in the early embryonic stages and/or the addition and proliferation in the late embryonic and postnatal development. Further developmental and lineage tracing studies will be required to answer these questions.

In addition to its regulation on the function and number of corneal ResMϕ at a steady-state, our data demonstrated that miR-183/96/182 imposes a significant impact on the dynamics of Csf1r-EGFP^+^ myeloid-derived MPS cells, which include both ResMϕ and DCs, in response to bacterial infection. The ResMϕ or leukocyte disappearance reaction has been reported in peritoneal and plural cavities since 1960s; however, the mechanisms and functional significance of this reaction remains largely elusive (51, 78). Recently, a similar phenomenon was shown in the cornea in a corneal epithelial abrasion mouse model, which suggested that dynamic interplay of resident and infiltrating Mϕ plays an important role in corneal wound healing by balancing the inflammatory and wound healing responses (53). In the current study, we directly illustrated that alter P. *aeruginosa* infection, more than 80% of the Csf1r-EGFP^+^ corneal ResMϕ and DCs disappeared in both ko and wt controls at 3 hpi before the involvement of infiltrating immune cells, suggesting that miR-183/96/182 does not directly regulate the initial disappearance phase of ResMϕ and DCs. At 6 hpi, the Csf1r-EGFP^+^ MPS cells in the infected cornea of ko mice were a significantly increased when compared with the wt controls. However, because the circulating MPS cells have infiltrated to the infected cornea at this stage, the increased Csf1r-EGFP^+^ cells most possibly represent infiltrating myeloid cells, suggesting that miR-183/96/182 modulates the infiltration of circulating MPS cells in response to *P. aeruginosa* infection. We hypothesize that the early increased production of IL-17f by corneal ResMϕ and other proinflammatory cytokine/chemokines (e.g., Il-1β and Cxcl2) contribute to this effect. An indepth fate mapping system that definitively distinguishes circulating Mϕ and ResMϕ (79) and intravital live imaging will help uncover the mechanisms of the Mϕ disappearance reaction and dissect the roles of miR-183/96/182 in the dynamic interplay of ResMϕ and infiltrating leukocytes in bacterial keratitis.

The cornea interfaces the eye and the external environment and is the start of the vision (55, 56). These unique functions of the cornea (4, 55–57) demand special features of its resident immune cells to be able to mount swift inflammatory response against microbial infection or other injuries and meanwhile contain and avoid excessive inflammation and promote restoration of the integrity and functions of the cornea and endow the eye with immune privilege (4, 55, 56). Our data demonstrated that corneal ResMϕ are, simultaneously, innate IL-17f- and IL-10-producing cells. This double-edged function of corneal ResMϕ with both proinflammatory and immune-regulatory potential may be particularly important for the maintenance and homeostasis of the cornea and the establishment of immune privilege of the eye. Dual regulations of miR-183/96/182 on the function and the number of corneal ResMϕ place miR-183/96/182 at the center of corneal innate immunity. These data also suggest that miR-183/96/182 is a viable therapeutic target for the treatment of *P. aeruginosa* and possibly other microbial keratitis by modulating the corneal innate immunity. Because various approaches have been developed to enhance or knock down the functions of miRNAs in vivo (80, 81), and the cornea is one of the most accessible location for drug administration, it is warranted to test the therapeutic potential of topical application of anti-miR-183/96/182 (80) in *P. aeruginosa* keratitis.

## Supplementary Material

1

## Figures and Tables

**TABLE I.** Number of resident innate immune cells per cornea by flatmount immunofluorescence

	CD45^+^ (*n* = 4)		CD11b^+^ (*n* = 3)		F4/80^+^ (*n* = 4)	
	Average	SEM	Average	SEM	Average	SEM
wt	621	33	664	52	643	31
ko	1133	48	1182	47	1127	24
Fold (ko/wt)	1.82		1.78		1.75	

**TABLE II.** Csf1r-EGFP^+^ cell counts per cornea (8–12 wk old)

	Naive (*n* = 7)		3 hpi (Infected Eye) (*n* = 3)		6 hpi (Infected Eye) (*n* = 3)		3 hpi (Contralateral Eye) (*n* = 3)		6 hpi (Contralateral Eye) (*n* = 3)	
	Average	SEM	Average	SEM	Average	SEM	Average	SEM	Average	SEM
wt	1084	45	197	22	1339	367	213	63	511	170
ko	1672	71	300	19	3633	848	326	135	807	229
	Naive	SEM	3 hpi	SEM	6 hpi	SEM	3 hpi	SEM	6 hpi	SEM
Percentage normalized to naive ko cornea										
wt	65	3	12	1	80	22	13	4	31	10
ko	100	4	18	1	217	51	19	8	48	14
Percentage normalized to ko or wt’s own naive cornea										
wt	100	4	18	2	124	34	20	6	47	16
ko	100	4	18	1	217	51	19	8	48	14

## Abbreviations used in this article:

AF546: Alexa Fluor 546
ATCC: American Type Culture Collection
CRIC: corneal resident immune cell
DC: dendritic cell
GT: gene-trap
hpi: hour postinfection
ko: knockout
Mϕ: macrophage
MC: monocyte
miR-183/96/182: miR-183/96/182 cluster
miRNA: microRNA
MOI: multiplicity of infection
MPS: mononuclear phagocyte system
P21: postnatal 21-day-old
PBS+: PBS plus 2 mM MgCl_2_
PFA: paraformaldehyde
qRT-PCR: quantitative RT-PCR
ResMϕ: resident Mϕ
RT: room temperature
scr: oligonucleotide duplex with scrambled sequence
wt: wild-type
