# Supplementary material for: The miR-183/96/182 Cluster Regulates the Functions of Corneal Resident Macrophages
Source: Immunohorizons. Author manuscript; Available in PMC 2021 Feb 18. (PMC7891884; doi:10.4049/immunohorizons.2000091)
Supplement: 1 [file NIHMS1666856-supplement-1.pdf]

Supplemental Information for

The miR-183/96/182 cluster regulates the functions of corneal resident macrophages

Ardian Coku[*], Sharon A. McClellan[*], Eric Van Buren[†], Jessica B. Back[†], Linda D. Hazlett[*], Shunbin Xu[*‡]

[*] Department of Ophthalmology, Visual and Anatomical Sciences; [†] Department of Oncology, School of Medicine, Wayne State University, Detroit, Michigan

[‡] **Corresponding author**: Shunbin Xu, Department of Ophthalmology, Visual and Anatomical Sciences, Wayne State University School of Medicine, 540 E. Canfield Street, Detroit, MI 48201, USA. Tel: 313-577-0127. Fax: 313-577-3125 sxu@med.wayne.edu

This PDF file includes:

Supplemental figures 1-6

Supplemental tables 1, 2

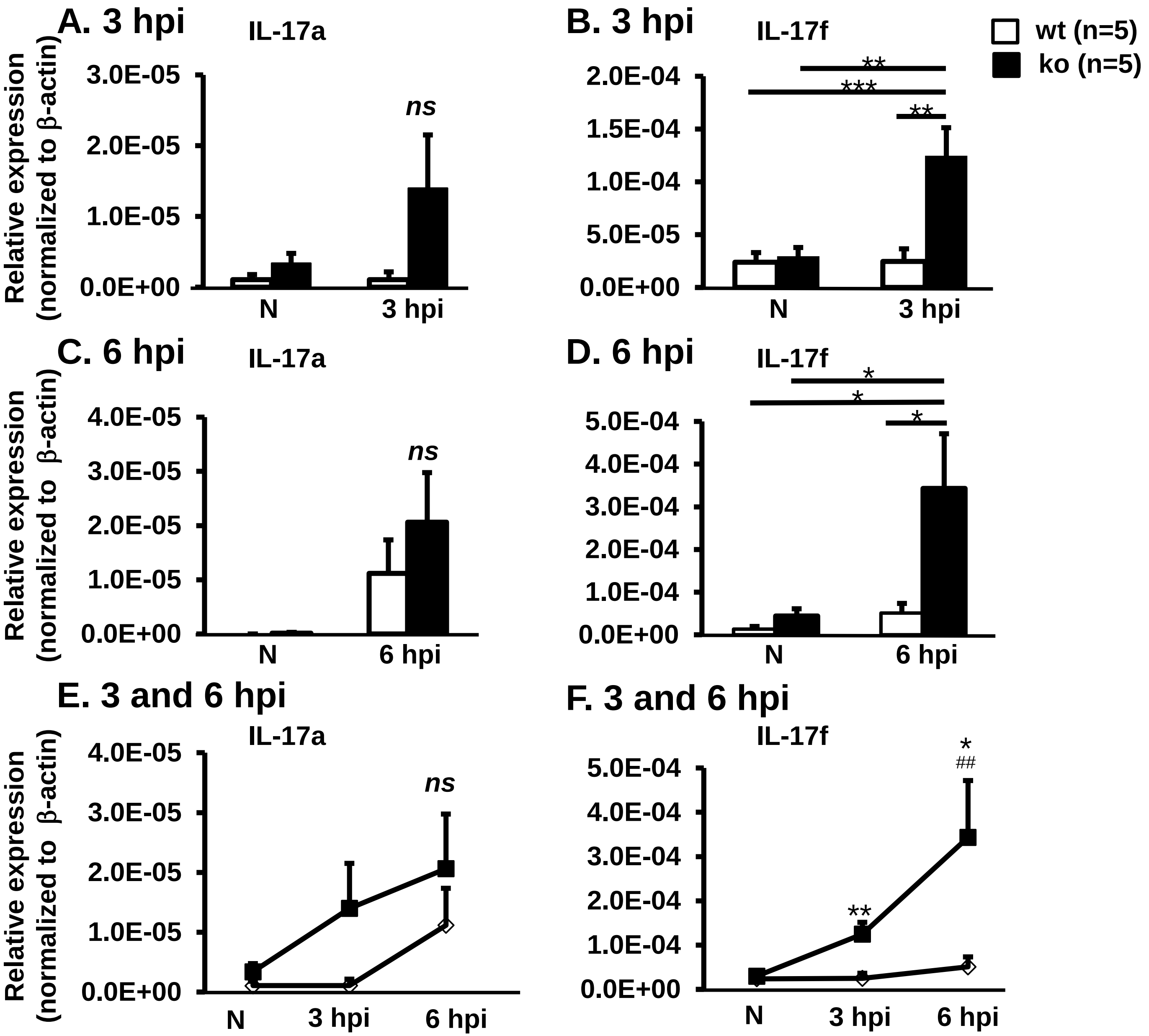

Supplemental Figure 1. Dynamic changes of miR-17a and miR-17f expression at 3 and 6 hpi. qRT-PCR of IL-17a (A,C,E) and IL-17f (**B,D,F**) in the corneas of young adult (8-12 weeks old) wt and ko mice at 3 (**A,B**) and 6 hpi (**C,D**) of PA infection. n=5/group. N: non-infected contralateral eyes. * and **: adjusted *p*<0.05 and *p*<0.01, respectively, in comparison of ko vs. wt. ## : adjusted *p*<0.01 in comparison between 3 and 6 hpi. n.s.: not statistically significant.

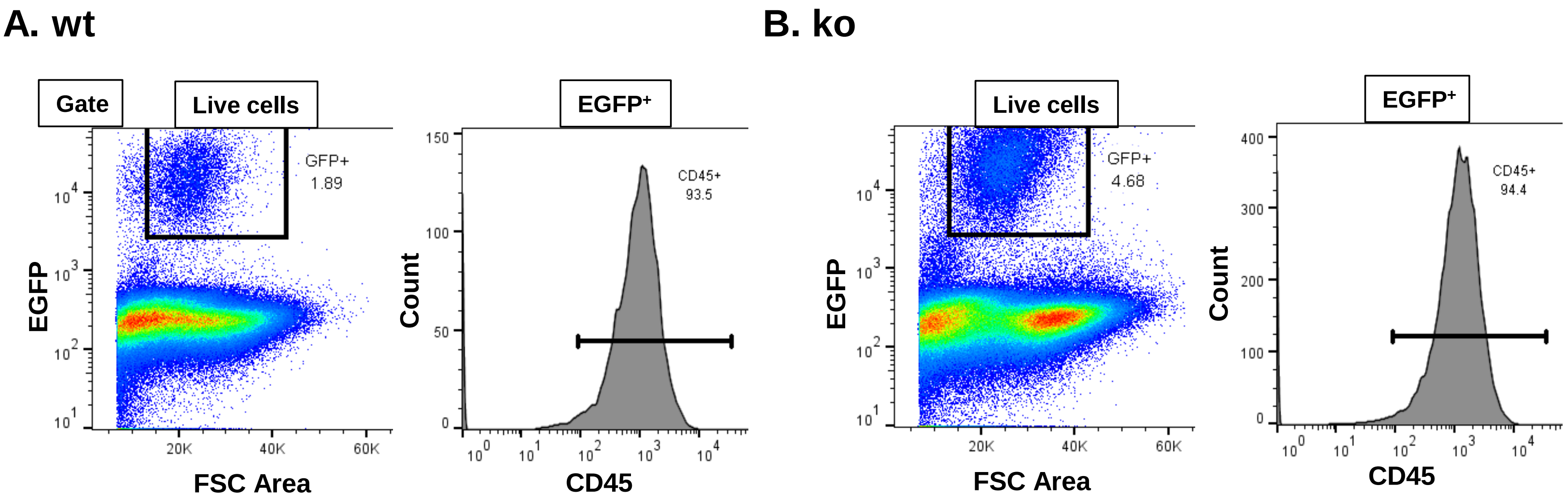

Supplemental Figure 2. >90% of the EGFP$^+$ cells are CD45$^+$ in the cornea of both miR-183/96/182 ko and wt control mice.

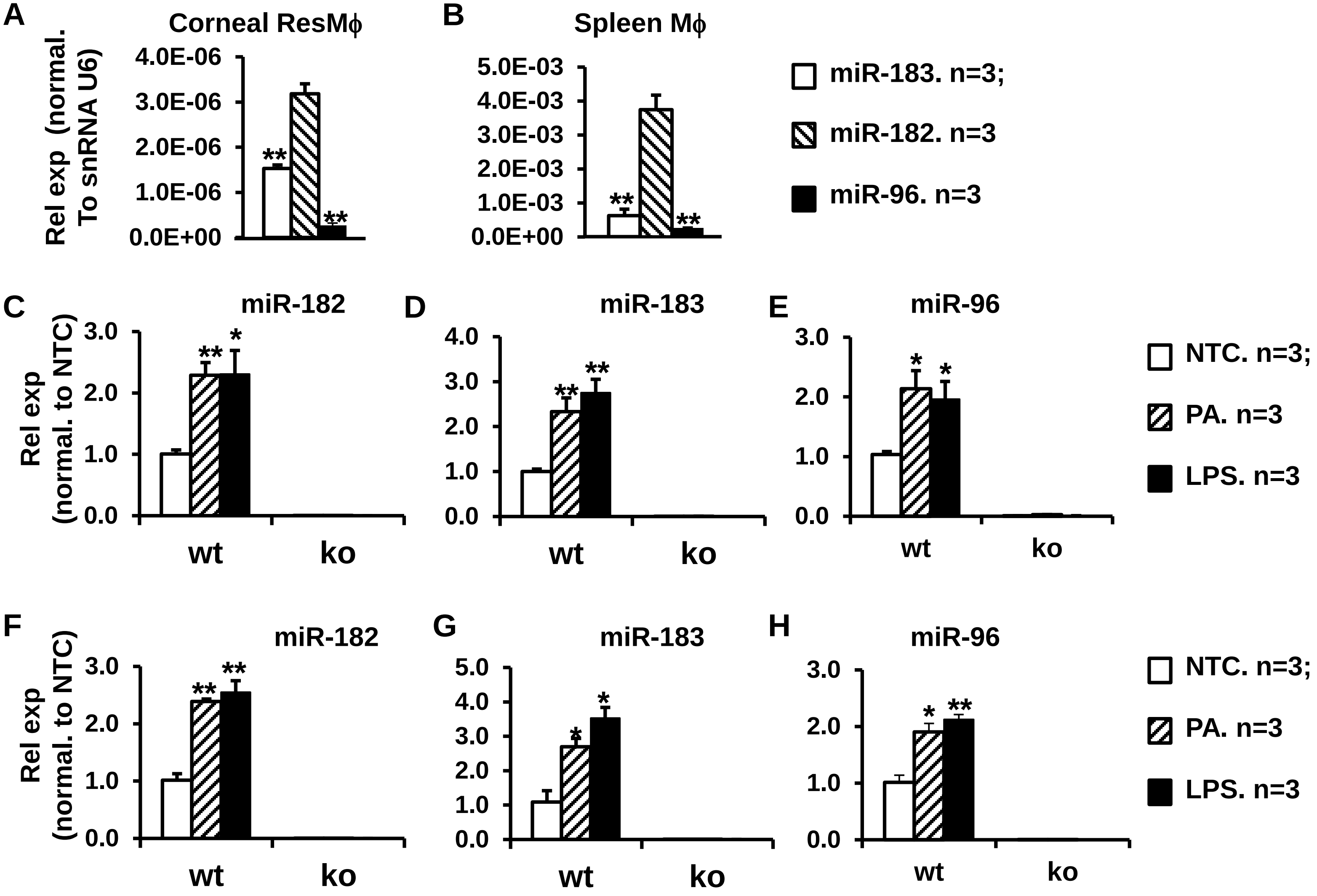

Supplemental Figure 3. miR-183/96/182 expression in corneal ResMϕ (A, C-E) and spleen Mϕ (B, F-H) in response to PA and LPS treatment. A, B: non-treated FACS-sorted $CD45^{+}CD11b^{+}F4/80^{+}$ corneal ResMϕ (A) and spleen Mϕ. **: *p*<0.01 vs. miR-182. (C-E) corneal ResMϕ and (F-G) spleen Mϕ in response to 6-hour treatment of PA (MOI=5) or LPS (100 ng/ml). Relative expression levels (Rel exp) were normalized to snRNA U6 (A, B) or to the non-treated controls (NTC) (C-H). n=3/group. *: *p*<0.05; **: *p*<0.01 vs. wt NTC.

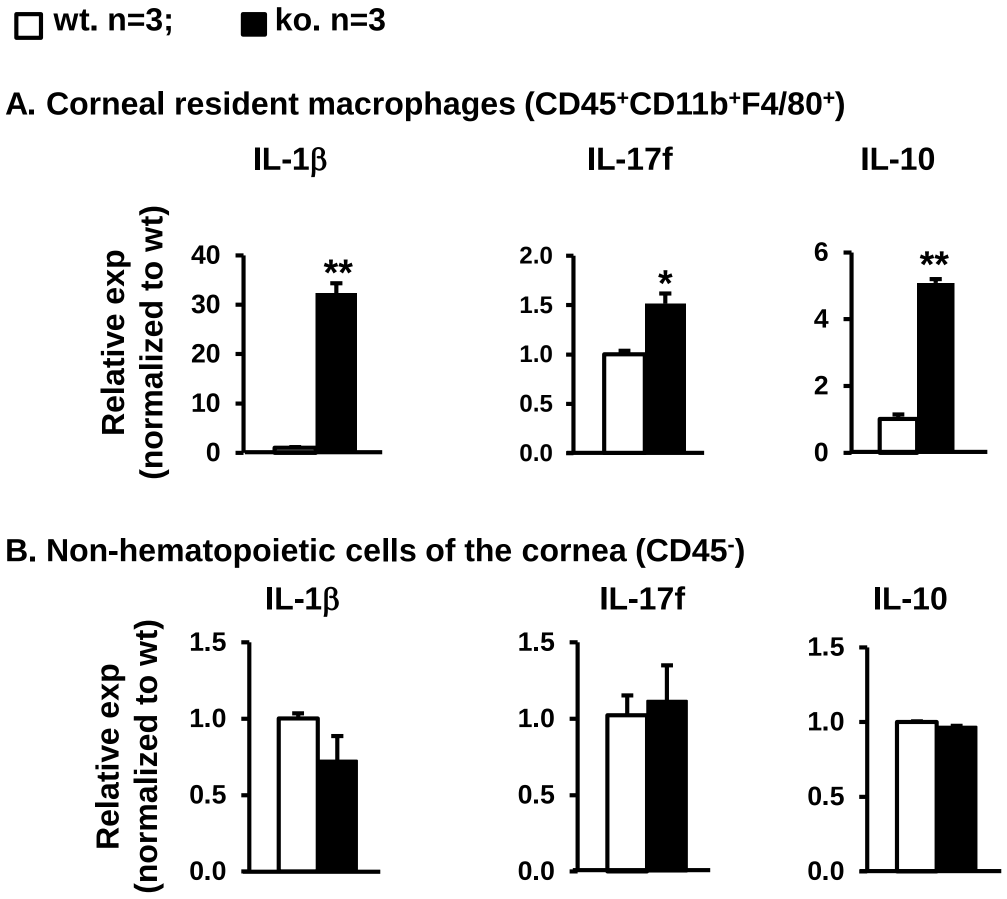

Supplemental Figure 4. IL-1β, IL-17f and IL-10 are simultaneously upregulated in corneal ResMϕ ($CD45^+CD11b^+F4/80^+$) (**A**) but not in the $CD45^-$ non-hematopoietic cells (**B**) of naïve miR-183/96/182 ko mice. qRT-PCR of IL-1β, IL-17f and IL-10. n=3/group. *: $p<0.05$; **: $p<0.01$.

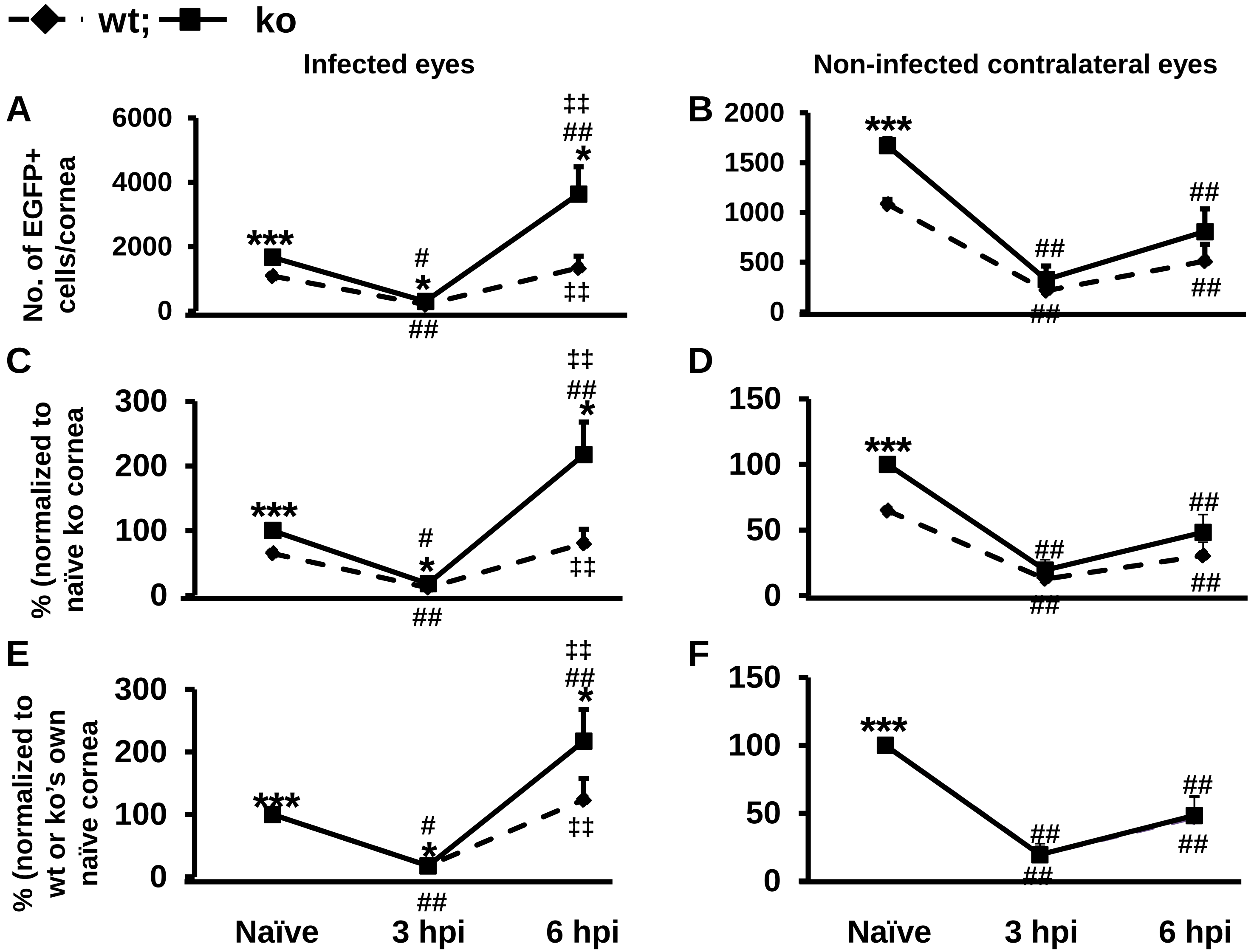

Supplemental Figure 5. Csf1r-EGFP⁺ MPS cells in the cornea have different dynamics in response to PA infection in miR-183/96/182 ko mice. Csf1r-GFP⁺ cell counts in corneal stroma flatmount of 8-12 weeks old miR-183/96/182 ko and age-matched wt controls at 3 hpi (n=3/genotype) and 6 hpi (n=3/genotype) in comparison to naïve mouse cornea (n=7/genotype). **A,C, E**: infected eyes; **B,D,F**: non-infected contralateral eyes. * and ***: $p<0.05$ and $p<0.001$, respectively, in comparison of ko vs. wt. # and ## : $p<0.05$ and $p<0.01$, respectively, in comparison of 3 or 6 hpi vs. naïve mice; ‡ and ‡‡: $p<0.05$ and $p<0.01$, respectively, in comparison of numbers at 3 vs. 6 hpi in ko (above the curves) or wt control mice (below the curves).

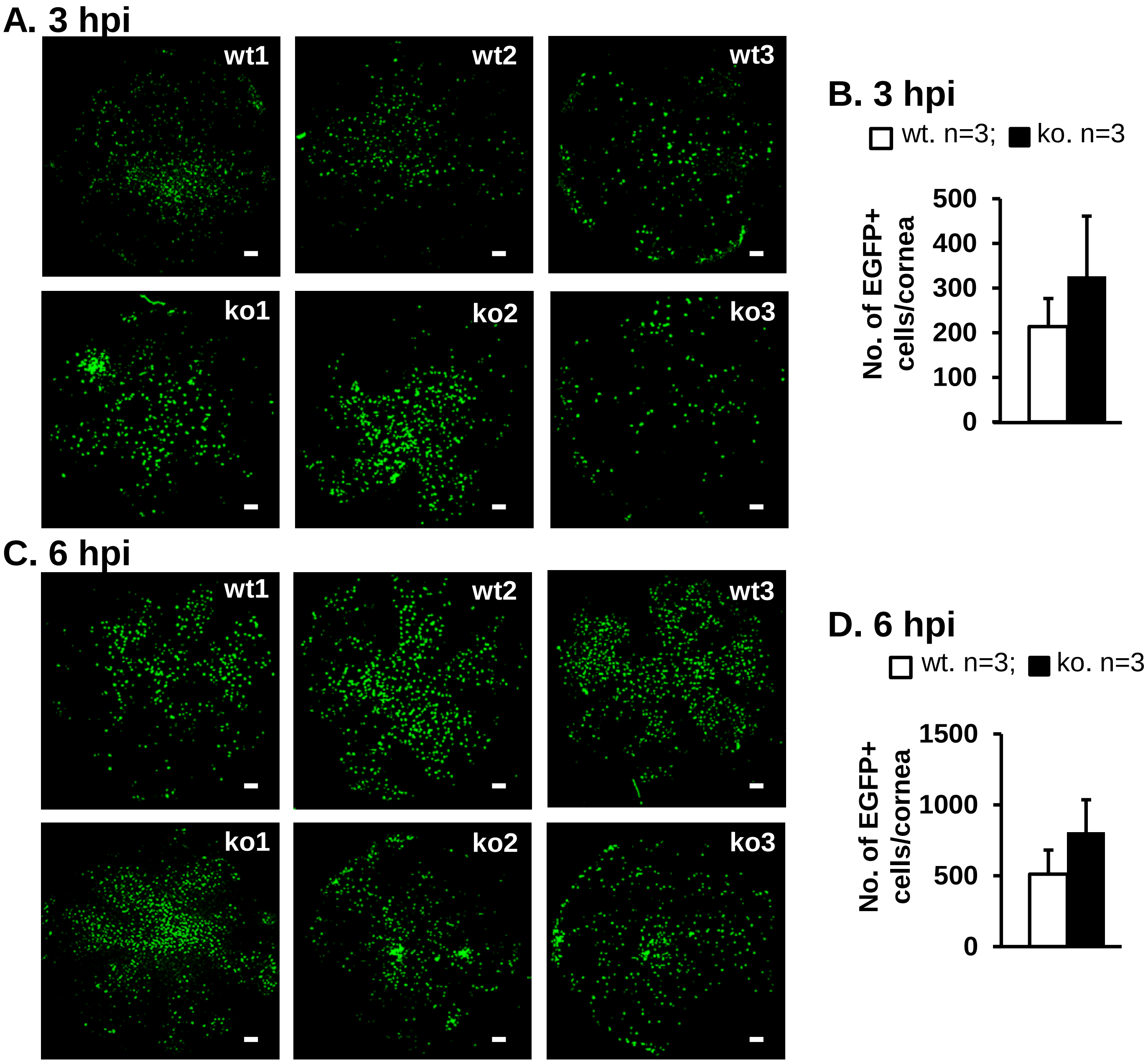

Supplemental Figure 6. Dynamics of Csf1r-EGFP+ MPS cells in the non-infected contralateral eyes of PA-infected mice at 3 and 6 hpi. Csf1r-GFP+ cell counts in corneal stromal flatmount of 8-12 weeks old miR-183/96/182 ko mice and age-matched wt controls (n=3/genotype).

Supplemental table 1. Custome RT2 PCR array 1 (Cat No. 330171 CLAIM25224)

Position	UniGene	GenBank	Symbol	Description
A01	Mm.2209	NM_013488	Cd4	CD4 antigen
A02	Mm.42044	NM_017480	Icos	Inducible T-cell co-stimulator
A03	Mm.322843	NM_020583	Isg20	Interferon-stimulated protein
A04	Mm.843	NM_008511	Lrmp	Lymphoid-restricted membrane protein
A05	Mm.4922	NM_009969	Csf2	Colony stimulating factor 2 (granulocyte-
A06	Mm.1238	NM_009971	Csf3	Colony stimulating factor 3 (granulocyte)
A07	Mm.240327	NM_008337	Ifng	Interferon gamma
A08	Mm.874	NM_010548	Il10	Interleukin 10
A09	Mm.239707	NM_001303244	Il12b	Interleukin 12b
A10	Mm.1284	NM_008355	Il13	Interleukin 13
A11	Mm.4392	NM_008357	Il15	Interleukin 15
A12	Mm.5419	NM_010552	Il17a	Interleukin 17A
B01	Mm.222808	NM_145834	Il17c	Interleukin 17C
B02	Mm.390726	NM_145837	Il17d	Interleukin 17D
B03	Mm.222807	NM_145856	Il17f	Interleukin 17F
B04	Mm.1410	NM_008360	Il18	Interleukin 18
B05	Mm.222830	NM_008361	Il1b	Interleukin 1 beta
B06	Mm.14190	NM_008366	Il2	Interleukin 2
B07	Mm.393490	NM_021782	Il21	Interleukin 21
B08	Mm.103585	NM_016971	Il22	Interleukin 22
B09	Mm.125482	NM_031252	Il23a	Interleukin 23, alpha subunit p19
B10	Mm.90154	NM_080729	Il25	Interleukin 25
B11	Mm.222632	NM_145636	Il27	Interleukin 27
B12	Mm.983	NM_010556	Il3	Interleukin 3
C01	Mm.276360	NM_021283	Il4	Interleukin 4
C02	Mm.4461	NM_010558	Il5	Interleukin 5
C03	Mm.1019	NM_001314054	Il6	Interleukin 6
C04	Mm.3006	NM_008373	Il9	Interleukin 9
C05	Mm.248380	NM_011577	Tgfb1	Transforming growth factor, beta 1
C06	Mm.3992	NM_009368	Tgfb3	Transforming growth factor, beta 3
C07	Mm.1293	NM_013693	Tnf	Tumor necrosis factor
C08	Mm.1283	NM_011329	Ccl1	Chemokine (C-C motif) ligand 1
C09	Mm.290320	NM_011333	Ccl2	Chemokine (C-C motif) ligand 2
C10	Mm.1282	NM_011337	Ccl3	Chemokine (C-C motif) ligand 3
C11	Mm.244263	NM_013652	Ccl4	Chemokine (C-C motif) ligand 4
C12	Mm.284248	NM_013653	Ccl5	Chemokine (C-C motif) ligand 5
D01	Mm.116739	NM_016960	Ccl20	Chemokine (C-C motif) ligand 20
D02	Mm.12895	NM_009137	Ccl22	Chemokine (C-C motif) ligand 22
D03	Mm.341574	NM_013654	Ccl7	Chemokine (C-C motif) ligand 7
D04	Mm.103711	NM_009142	Cx3cl1	Chemokine (C-X3-C motif) ligand 1
D05	Mm.21013	NM_008176	Cxcl1	Chemokine (C-X-C motif) ligand 1
D06	Mm.303231	NM_021704	Cxcl12	Chemokine (C-X-C motif) ligand 12
D07	Mm.4979	NM_009140	Cxcl2	Chemokine (C-X-C motif) ligand 2
D08	Mm.244289	NM_203320	Cxcl3	Chemokine (C-X-C motif) ligand 3
D09	Mm.4660	NM_009141	Cxcl5	Chemokine (C-X-C motif) ligand 5
D10	Mm.6272	NM_009915	Ccr2	Chemokine (C-C motif) receptor 2
D11	Mm.1337	NM_009916	Ccr4	Chemokine (C-C motif) receptor 4
D12	Mm.8007	NM_009835	Ccr6	Chemokine (C-C motif) receptor 6
E01	Mm.896	NM_008362	Il1r1	Interleukin 1 receptor, type I
E02	Mm.731	NM_008353	Il12rb1	Interleukin 12 receptor, beta 1
E03	Mm.188337	NM_008354	Il12rb2	Interleukin 12 receptor, beta 2
E04	Mm.4481	NM_008359	Il17ra	Interleukin 17 receptor A
E05	Mm.269363	NM_019583	Il17rb	Interleukin 17 receptor B

E06	Mm.213397	NM_134159	Il17rc	Interleukin 17 receptor C
E07	Mm.131781	NM_145826	Il17re	Interleukin 17 receptor E
E08	Mm.221227	NM_144548	Il23r	Interleukin 23 receptor
E09	Mm.2856	NM_010559	Il6ra	Interleukin 6 receptor, alpha
E10	Mm.389	NM_008372	Il7r	Interleukin 7 receptor
E11	Mm.4861	NM_011616	Cd40lg	CD40 ligand
E12	Mm.439656	NM_009883	Cebpb	CCAAT/enhancer binding protein (C/EBP),
F01	Mm.239516	NM_020008	Clec7a	C-type lectin domain family 7, member a
F02	Mm.182291	NM_054039	Foxp3	Forkhead box P3
F03	Mm.313866	NM_008091	Gata3	GATA binding protein 3
F04	Mm.4677	NM_013674	Irf4	Interferon regulatory factor 4
F05	Mm.289657	NM_146145	Jak1	Janus kinase 1
F06	Mm.275839	NM_008413	Jak2	Janus kinase 2
F07	Mm.4993	NM_010809	Mmp3	Matrix metallopeptidase 3
F08	Mm.4406	NM_013599	Mmp9	Matrix metallopeptidase 9
F09	Mm.213003	NM_010851	Myd88	Myeloid differentiation primary response gene
F10	Mm.116802	NM_010899	Nfatc2	Nuclear factor of activated T-cells,
F11	Mm.256765	NM_008689	Nfkb1	Nuclear factor of kappa light polypeptide
F12	Mm.378450	NM_013646	Rora	RAR-related orphan receptor alpha
G01	Mm.4372	NM_011281	Rorc	RAR-related orphan receptor gamma
G02	Mm.4081	NM_009821	Runx1	Runt related transcription factor 1
G03	Mm.982	NM_007901	S1pr1	Sphingosine-1-phosphate receptor 1
G04	Mm.130	NM_009896	Socs1	Suppressor of cytokine signaling 1
G05	Mm.3468	NM_007707	Socs3	Suppressor of cytokine signaling 3
G06	Mm.473190	NM_011486	Stat3	Signal transducer and activator of
G07	Mm.1550	NM_011487	Stat4	Signal transducer and activator of
G08	Mm.277403	NM_011488	Stat5a	Signal transducer and activator of
G09	Mm.121721	NM_009284	Stat6	Signal transducer and activator of
G10	Mm.375031	NM_011518	Syk	Spleen tyrosine kinase
G11	Mm.94519	NM_019507	Tbx21	T-box 21
G12	Mm.292729	NM_009424	Traf6	Tnf receptor-associated factor 6
H01	Mm.1051	NM_009807	Casp1	Caspase 1
H02	Mm.14874	NM_013542	Gzmb	Granzyme B
H03	Mm.4528	NM_008479	Lag3	Lymphocyte-activation gene 3
H04	Mm.248615	NM_010705	Lgals3	Lectin, galactose binding, soluble 3
H05	Mm.309092	NM_008084	Gapdh	Glyceraldehyde-3-phosphate dehydrogenase
H06	Mm.328431	NM_007393	Actb	Actin, beta
H07	Mm.163	NM_009735	B2m	Beta-2 microglobulin
H08	Mm.328431	NM_007393	Actb	Actin, beta
H09	Mm.328431	NM_007393	Actb	Actin, beta
H10	N/A	SA_00104	RTC	Reverse Transcription Control
H11	N/A	SA_00103	PPC	Positive PCR Control
H12	N/A	SA_00106	MGDC	Mouse Genomic DNA Contamination

Supplemental table 2. Custome RT2 PCR array 2 - focused panel (Cat No. 330171 CLAIM30394)

Position	UniGene	GenBank	Symbol	Description
A01	Mm.874	NM_010548	Il10	Interleukin 10
A02	Mm.5419	NM_010552	Il17a	Interleukin 17A
A03	Mm.390726	NM_145837	Il17d	Interleukin 17D
B01	Mm.222807	NM_145856	Il17f	Interleukin 17F
B02	Mm.222830	NM_008361	Il1b	Interleukin 1 beta
B03	Mm.125482	NM_031252	Il23a	Interleukin 23, alpha subunit p19
C01	Mm.4461	NM_010558	Il5	Interleukin 5
C02	Mm.248380	NM_011577	Tgfb1	Transforming growth factor, beta 1
C03	Mm.290320	NM_011333	Ccl2	Chemokine (C-C motif) ligand 2
D01	Mm.284248	NM_013653	Ccl5	Chemokine (C-C motif) ligand 5
D02	Mm.4979	NM_009140	Cxcl2	Chemokine (C-X-C motif) ligand 2
D03	Mm.6272	NM_009915	Ccr2	Chemokine (C-C motif) receptor 2
E01	Mm.188337	NM_008354	Il12rb2	Interleukin 12 receptor, beta 2
E02	Mm.131781	NM_145826	Il17re	Interleukin 17 receptor E
E03	Mm.239516	NM_020008	Clec7a	C-type lectin domain family 7, member a
F01	Mm.275839	NM_008413	Jak2	Janus kinase 2
F02	Mm.4372	NM_011281	Rorc	RAR-related orphan receptor gamma
F03	Mm.4081	NM_009821	Runx1	Runt related transcription factor 1
G01	Mm.121721	NM_009284	Stat6	Signal transducer and activator of transcription 6
G02	Hs.592355	NM_002046	GAPDH	Glyceraldehyde-3-phosphate dehydrogenase
G03	Mm.328431	NM_007393	Actb	Actin, beta
H01	N/A	SA_00104	RTC	Reverse Transcription Control
H02	N/A	SA_00103	PPC	Positive PCR Control
H03	N/A	SA_00106	MGDC	Mouse Genomic DNA Contamination
A04	Mm.874	NM_010548	Il10	Interleukin 10
A05	Mm.5419	NM_010552	Il17a	Interleukin 17A
A06	Mm.390726	NM_145837	Il17d	Interleukin 17D
B04	Mm.222807	NM_145856	Il17f	Interleukin 17F
B05	Mm.222830	NM_008361	Il1b	Interleukin 1 beta
B06	Mm.125482	NM_031252	Il23a	Interleukin 23, alpha subunit p19
C04	Mm.4461	NM_010558	Il5	Interleukin 5
C05	Mm.248380	NM_011577	Tgfb1	Transforming growth factor, beta 1
C06	Mm.290320	NM_011333	Ccl2	Chemokine (C-C motif) ligand 2
D04	Mm.284248	NM_013653	Ccl5	Chemokine (C-C motif) ligand 5
D05	Mm.4979	NM_009140	Cxcl2	Chemokine (C-X-C motif) ligand 2
D06	Mm.6272	NM_009915	Ccr2	Chemokine (C-C motif) receptor 2
E04	Mm.188337	NM_008354	Il12rb2	Interleukin 12 receptor, beta 2
E05	Mm.131781	NM_145826	Il17re	Interleukin 17 receptor E
E06	Mm.239516	NM_020008	Clec7a	C-type lectin domain family 7, member a
F04	Mm.275839	NM_008413	Jak2	Janus kinase 2
F05	Mm.4372	NM_011281	Rorc	RAR-related orphan receptor gamma
F06	Mm.4081	NM_009821	Runx1	Runt related transcription factor 1
G04	Mm.121721	NM_009284	Stat6	Signal transducer and activator of transcription 6
G05	Hs.592355	NM_002046	GAPDH	Glyceraldehyde-3-phosphate dehydrogenase
G06	Mm.328431	NM_007393	Actb	Actin, beta
H04	N/A	SA_00104	RTC	Reverse Transcription Control
H05	N/A	SA_00103	PPC	Positive PCR Control
H06	N/A	SA_00106	MGDC	Mouse Genomic DNA Contamination
A07	Mm.874	NM_010548	Il10	Interleukin 10
A08	Mm.5419	NM_010552	Il17a	Interleukin 17A

A09	Mm.390726	NM_145837	Il17d	Interleukin 17D
B07	Mm.222807	NM_145856	Il17f	Interleukin 17F
B08	Mm.222830	NM_008361	Il1b	Interleukin 1 beta
B09	Mm.125482	NM_031252	Il23a	Interleukin 23, alpha subunit p19
C07	Mm.4461	NM_010558	Il5	Interleukin 5
C08	Mm.248380	NM_011577	Tgfb1	Transforming growth factor, beta 1
C09	Mm.290320	NM_011333	Ccl2	Chemokine (C-C motif) ligand 2
D07	Mm.284248	NM_013653	Ccl5	Chemokine (C-C motif) ligand 5
D08	Mm.4979	NM_009140	Cxcl2	Chemokine (C-X-C motif) ligand 2
D09	Mm.6272	NM_009915	Ccr2	Chemokine (C-C motif) receptor 2
E07	Mm.188337	NM_008354	Il12rb2	Interleukin 12 receptor, beta 2
E08	Mm.131781	NM_145826	Il17re	Interleukin 17 receptor E
E09	Mm.239516	NM_020008	Clec7a	C-type lectin domain family 7, member a
F07	Mm.275839	NM_008413	Jak2	Janus kinase 2
F08	Mm.4372	NM_011281	Rorc	RAR-related orphan receptor gamma
F09	Mm.4081	NM_009821	Runx1	Runt related transcription factor 1
G07	Mm.121721	NM_009284	Stat6	Signal transducer and activator of transcription 6
G08	Hs.592355	NM_002046	GAPDH	Glyceraldehyde-3-phosphate dehydrogenase
G09	Mm.328431	NM_007393	Actb	Actin, beta
H07	N/A	SA_00104	RTC	Reverse Transcription Control
H08	N/A	SA_00103	PPC	Positive PCR Control
H09	N/A	SA_00106	MGDC	Mouse Genomic DNA Contamination
A10	Mm.874	NM_010548	Il10	Interleukin 10
A11	Mm.5419	NM_010552	Il17a	Interleukin 17A
A12	Mm.390726	NM_145837	Il17d	Interleukin 17D
B10	Mm.222807	NM_145856	Il17f	Interleukin 17F
B11	Mm.222830	NM_008361	Il1b	Interleukin 1 beta
B12	Mm.125482	NM_031252	Il23a	Interleukin 23, alpha subunit p19
C10	Mm.4461	NM_010558	Il5	Interleukin 5
C11	Mm.248380	NM_011577	Tgfb1	Transforming growth factor, beta 1
C12	Mm.290320	NM_011333	Ccl2	Chemokine (C-C motif) ligand 2
D10	Mm.284248	NM_013653	Ccl5	Chemokine (C-C motif) ligand 5
D11	Mm.4979	NM_009140	Cxcl2	Chemokine (C-X-C motif) ligand 2
D12	Mm.6272	NM_009915	Ccr2	Chemokine (C-C motif) receptor 2
E10	Mm.188337	NM_008354	Il12rb2	Interleukin 12 receptor, beta 2
E11	Mm.131781	NM_145826	Il17re	Interleukin 17 receptor E
E12	Mm.239516	NM_020008	Clec7a	C-type lectin domain family 7, member a
F10	Mm.275839	NM_008413	Jak2	Janus kinase 2
F11	Mm.4372	NM_011281	Rorc	RAR-related orphan receptor gamma
F12	Mm.4081	NM_009821	Runx1	Runt related transcription factor 1
G10	Mm.121721	NM_009284	Stat6	Signal transducer and activator of transcription 6
G11	Hs.592355	NM_002046	GAPDH	Glyceraldehyde-3-phosphate dehydrogenase
G12	Mm.328431	NM_007393	Actb	Actin, beta
H10	N/A	SA_00104	RTC	Reverse Transcription Control
H11	N/A	SA_00103	PPC	Positive PCR Control
H12	N/A	SA_00106	MGDC	Mouse Genomic DNA Contamination
